# Mitochondrial Thermogenesis Can Trigger Heat Shock Response in the Nucleus

**DOI:** 10.1021/acscentsci.3c01589

**Published:** 2024-06-03

**Authors:** Myeong-Gyun Kang, Hwa-Ryeon Kim, Hee Yong Lee, Chulhwan Kwak, Hyewon Koh, Byoung Heon Kang, Jae-Seok Roe, Hyun-Woo Rhee

**Affiliations:** † Department of Chemistry, Seoul National University, Seoul 08826, Korea; ‡ Department of Biochemistry, Yonsei University, Seoul 03722, Korea; § Department of Biological Sciences, Ulsan National Institute of Science and Technology (UNIST), Ulsan 44919, Korea; ∥ School of Biological Sciences, Seoul National University, Seoul 08826, Korea

## Abstract

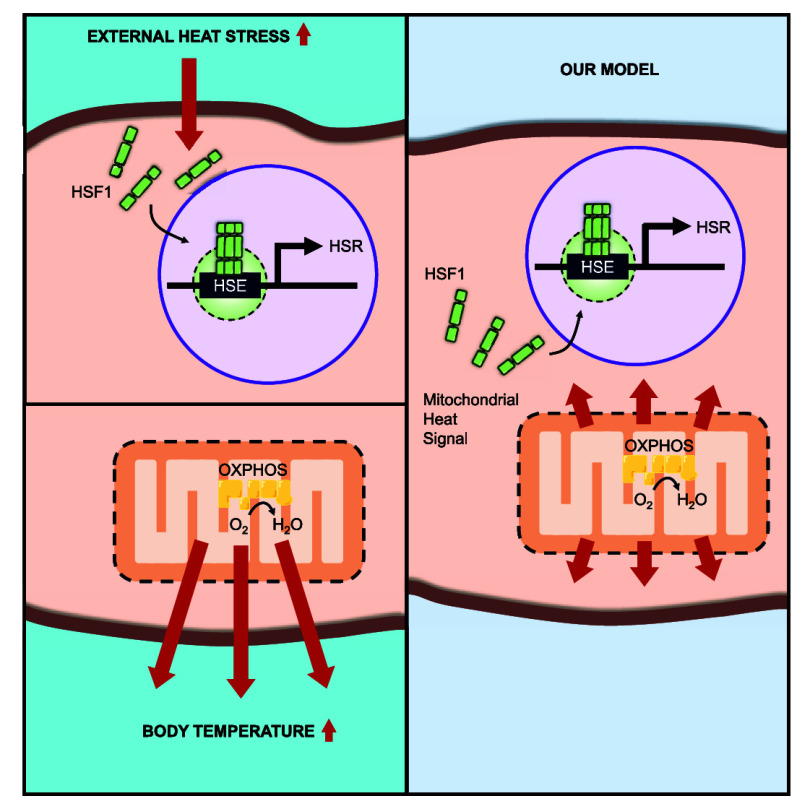

Mitochondrial thermogenesis is a process in which heat is generated by mitochondrial respiration. In living organisms, the thermogenic mechanisms that maintain body temperature have been studied extensively in fat cells with little knowledge on how mitochondrial heat may act beyond energy expenditure. Here, we highlight that the exothermic oxygen reduction reaction (Δ*H*_f_° = −286 kJ/mol) is the main source of the protonophore-induced mitochondrial thermogenesis, and this heat is conducted to other cellular organelles, including the nucleus. As a result, mitochondrial heat that reached the nucleus initiated the classical heat shock response, including the formation of nuclear stress granules and the localization of heat shock factor 1 (HSF1) to chromatin. Consequently, activated HSF1 increases the level of gene expression associated with the response to thermal stress in mammalian cells. Our results illustrate heat generated within the cells as a potential source of mitochondria-nucleus communication and expand our understanding of the biological functions of mitochondria in cell physiology.

## Abstract 2

Oxygen reduction reaction (ORR) is the exothermic reaction in mitochondria, and ORR-driven mitochondrial thermogenesis can induce the HSF1-mediated heat shock response in the nucleus.

## Introduction

Heat induces diverse biological events, including biochemical reactions (e.g., reaction equilibrium and reaction rates)^1,2^ and structural changes in proteins^3^ and lipid membranes.^4^ For this reason, living systems can precisely sense and respond to temperature changes.^5^ Under the external heat stress conditions, heat shock factor 1 (HSF1) senses the increase in temperature and undergoes a structural change that enables it to regulate the expression of heat shock proteins (HSPs) thereby promoting cell survival. Although HSF1 activation under external heat shock conditions was discovered more than 30 years ago,^6−9^ whether mammalian cells can produce their own heat to initiate an HSF1-mediated heat shock response remains unknown.

**Figure 1:**
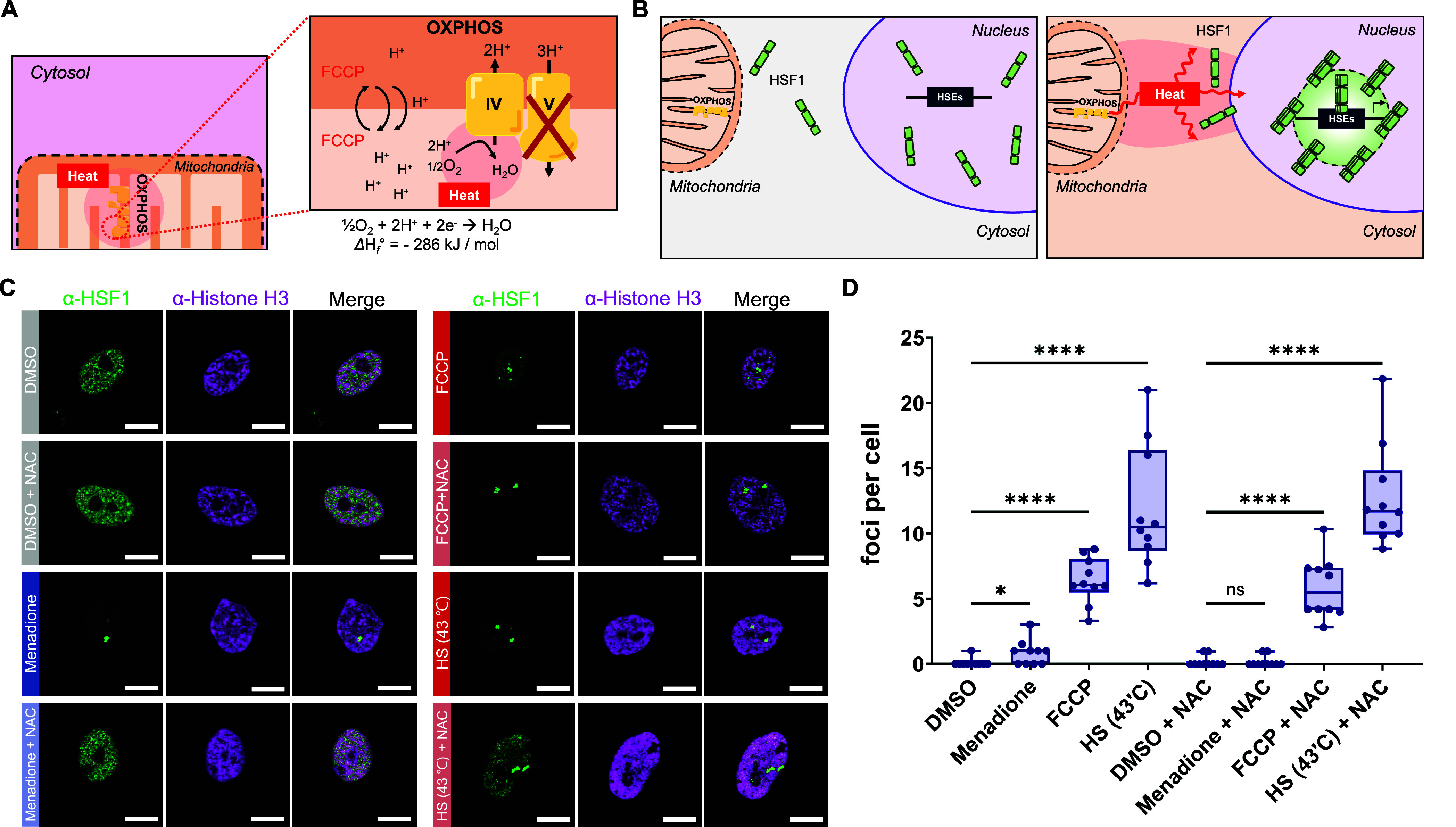
**Nuclear HSF1 activation by mitochondrial thermogenesis under FCCP treatment.** (**A**) Schematic representation of FCCP-induced exothermic mitochondrial oxygen reduction reaction (ORR). (**B**) Schematic representation of HSF1 activation via mitochondrial thermogenesis following FCCP treatment (HSEs: heat shock elements). (**C**) Confocal images of endogenous HSF1 (anti-HSF1) and Histone H3 (anti-Histone H3) activation in MCF10A cells incubated with either FCCP (100 μM, 1 h) or menadione (30 μM, 30 min) or subjected to heat shock (43 °C, 1 h) and with or without cotreatment with NAC (5 mM, 1 h). Scale bar = 10 μm. (**D**) The quantification of the number of foci per cell in both the control and various stress-induced cells corresponded to (C). In each condition, 50–100 cells were evaluated. Boxes indicate the quartiles, whiskers range from minimal to maximal values. Dots indicate individual data points plotted on the box. Statistical analysis was conducted using an unpaired two-tailed *t* test, and the significance level was denoted as (*p < 0.05, ****p < 0.0001, ns, not significant).

Mitochondrial thermogenesis is an intracellular event that actively generates heat to maintain the body temperature. In fat cell mitochondria, protons are directly imported into the mitochondrial matrix by the proton transporter protein (UCP1), not by ATP synthase,^10^ which is known to accelerate mitochondrial oxygen consumption.^11,12^ A similar event can be induced by treatment with mitochondrial uncoupling agents such as carbonyl cyanide *p*-(trifluoromethoxy)phenyl-hydrazone (FCCP), which can directly import protons to the mitochondrial matrix.^13^ Intriguingly, the FCCP-driven proton import to the mitochondrial matrix can also initiate mitochondrial thermogenesis, which was validated experimentally using several fluorescent thermometers that can precisely detect mitochondrial temperature increases in live cells.^14−22^ However, the biological importance and impact of the heat generated in mitochondria by changes in proton import remain uncertain. In this study, we highlighted that exothermic proton-coupled oxygen consumption reaction or oxygen reduction reaction (ORR, 1/2O_2_ + 2H^+^ + 2e^–^ → H_2_O, Δ*H*_f_° = −286 kJ/mol)^23,24^ (Figure 1A) is the main thermogenic source under the protonophore (i.e., FCCP) treatment by regulation of oxygen concentration and electron flows in the electron transport chain. Furthermore, we determined that this ORR-induced thermogenesis in mitochondria leads to thermal conduction to other organelles such as the nucleus. Under the same conditions, we observed that mitochondrial thermogenesis activated canonical nuclear heat shock response programs mediated by HSF1. Overall, our study reveals a cell-intrinsic mechanism that allows heat to actively convey biological signals from the mitochondria to the nucleus.

## Results

### Heat Shock Factor 1 Activation by Mitochondrial Thermogenesis

Biochemically, FCCP is a weak acid that facilitates reversible proton import from the mitochondrial intermembrane space (IMS) to the matrix, which in turn uncouples the electron transport chain and inhibits ATP synthesis.^25^ From a chemical perspective, this event can induce a highly exothermic oxygen reduction reaction (ORR) at mitochondrial complex IV (1/2O_2_ + 2H^+^ + 2e^–^ → H_2_O, Δ*H*_f_° = −286 kJ/mol)^23^ by providing essential protons for the reaction (Figure 1A). We confirmed that FCCP induced the mitochondrial uncoupling process by measuring the reduced mitochondrial membrane potential using the TMRE assay (Figure S1A,B). To verify whether other subcellular organelles are affected by heat produced by ORR, we measured that endoplasmic reticulum (ER) temperature was increased by the FCCP treatment with an ER-localized temperature-measuring fluorescent probe, ERthermAC (ETAC)^26^ (Figure S1C,D). Using a fluorescent polymeric thermometer (FDV) which is evenly distributed in the entire cellular area,^27−29^ we also observed that the fluorescence emission intensity ratio (FI_580_/FI_515_) of FDV corresponded to the ratio at the range from 38.9 to 40.0 °C under the FCCP treatment (Figure S1E,F). These results indicate that the mitochondrial-generated heat induced by FCCP can be transferred to the surrounding space and proximal organelles.

HSF1 is the fundamental heat-sensitive nuclear factor that can be activated with nuclear “foci” formation under heat stress.^30,31^ To determine whether the activity of HSF1 is regulated by mitochondrial heat (Figure 1B), we checked endogenous HSF1 localization by immunofluorescence imaging following treatment with FCCP. Under the FCCP treatment condition (100 μM, 1 h), endogenous HSF1 foci formation was clearly observed in the nucleus, and this pattern was comparable to that of HSF1 foci formation under external heat shock conditions (Figure 1C). As shown in the box plot (Figure 1D), a significant number of HSF1 foci were induced by FCCP (foci per cell: ∼6) and heat shock condition at 43 °C (foci per cell: ∼12), compared to DMSO-treated cells (foci per cell: 0). Additionally, the cell population with HSF1-GFP foci significantly increased in the FCCP-treated sample (∼70%) and in the heat shock (43 °C) induced sample (∼88%) compared to the control (Figure S2A,B).

**Figure 2:**
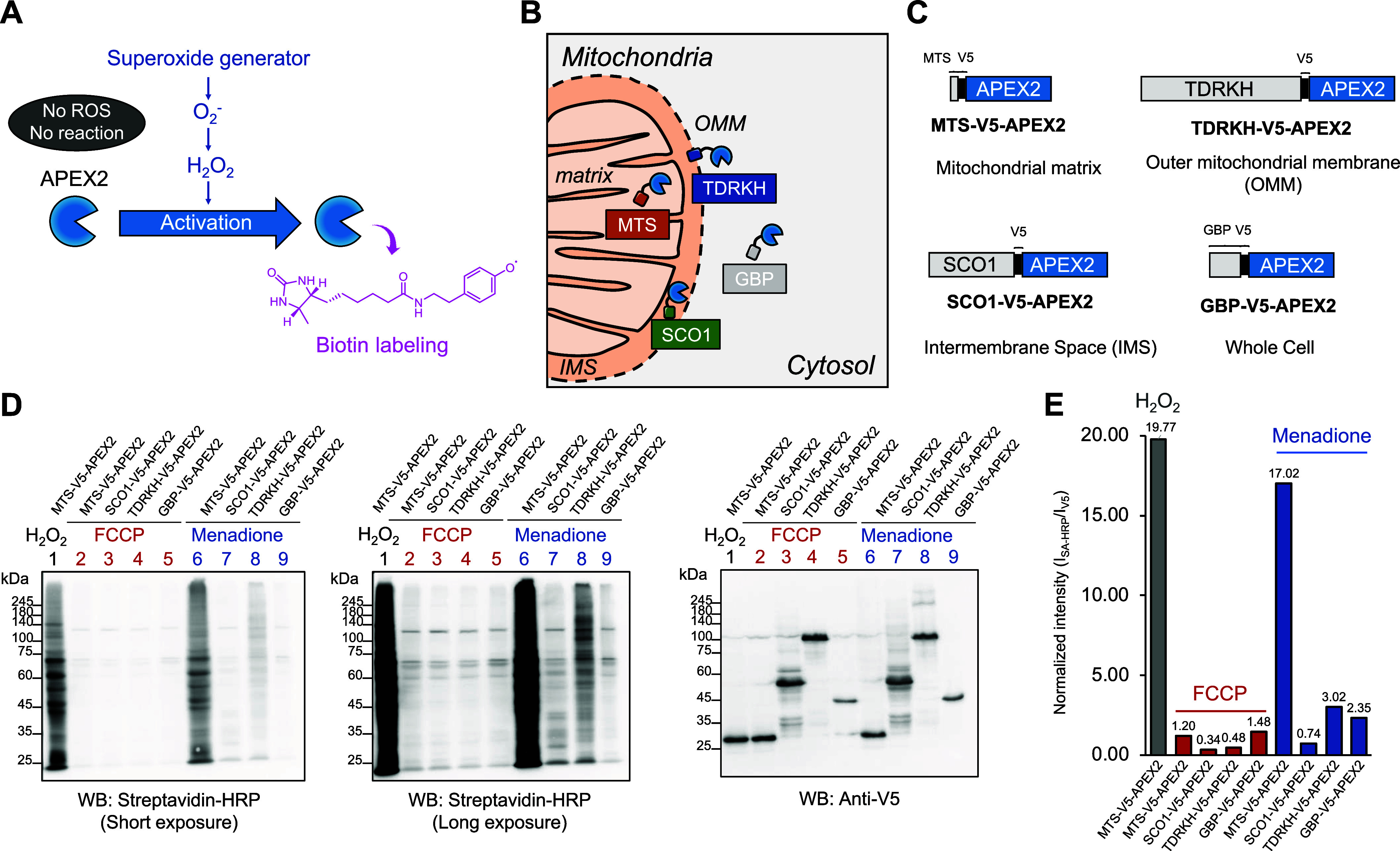
**FCCP treatment does not induce ROS generation.** (**A**) Schematic depiction of APEX-mediated recording of cellular ROS generation. (**B**) Graphical representation of the subcellular localization of APEX2 constructs used for the enzymatic recording of hydrogen peroxide generation: Matrix-V5-APEX2 (mitochondrial matrix), SCO1-V5-APEX2 (IMS), TDRKH-V5-APEX2 (OMM), and GBP-V5-APEX2 (whole cell). (**C**) Construct map of the APEX plasmids used in this study. (**D**) Streptavidin (SA) Western blot results of APEX-mediated biotinylating activity measurements after menadione (30 μM, 30 min) or FCCP (100 μM, 1 h) treatment. Anti-V5 Western blotting images of the same lysate are shown below. (**E**) Quantification plots of the relative band intensity after short exposure to streptavidin and anti-V5 antibodies.

Including heat, there are several factors that contribute to the process of HSF1 activation, such as intracellular pH changes,^32−34^ ROS generation,^35,36^ and proteotoxic stress.^37^ Hence, we decided to investigate whether mitochondrial heat is the primary factor triggering HSF1 foci formation with FCCP treatment. First of all, we excluded pH changes as factors in HSF1 activation because nuclear pH remained within the physiological range under FCCP treatment, as measured by pHluorin2^38^ (Figure S1G). To assess ROS generation under the FCCP treatment, we employed our recently developed system based on the ROS-dependent engineered ascorbate peroxidase (APEX) reaction^39^ (Figure 2A–C). As a result, the ROS production induced by FCCP was very low in all submitochondrial spaces compared to the well-established ROS-generating agent menadione^40^ (Figure 2D,E).

Notably, HSF1 has multiple activation modes either by heat stress or by ROS generation.^41,42^ We confirmed that HSF1 foci formation was induced by menadione without heat induction; however, the level of this ROS-induced HSF1 foci formation was much lower than that of FCCP (Figure 1D). In addition, menadione was significantly quenched by cotreatment with ROS quenching agent *N*-acetylcysteine (NAC, 5 mM) (Figure 1C,D and Figure S2A,B). In contrast, FCCP-driven HSF1 foci formation was minimally affected by cotreatment with the same concentration of NAC (5 mM), suggesting that ROS is unlikely attributed to HSF1 foci formation by FCCP (Figure 1C,D).

Next, we examined whether FCCP treatment activates HSF1 translocation to the nucleus via accumulation of mitochondrial precursor protein in the cytosol.^37^ For this assay, we monitored whether the mitochondrial import process of mitochondrial targeting sequence (MTS) conjugated dsRed (MTS-myc-dsRed) can be perturbed by our FCCP treatment condition (100 μM, 1 h). As a result, we could observe that both MTS-dsRed and endogenous HSPD1 (mitochondrial matrix resident protein) were well-targeted to the mitochondria under our FCCP treatment condition (Figure S3A,B). In the Western blot result, we could not detect the accumulation of precursor proteins of endogenous HSPD1 under the same FCCP treatment conditions (Figure S3C,D). These findings suggest that our short FCCP incubation time may not induce significant accumulation of mitochondrial precursor proteins in the cytosol. Additionally, we confirmed that HSF1 foci formation can be induced by FCCP treatment even under the condition of new protein synthesis being blocked by cycloheximide (CHX) or puromycin, while CHX or puromycin treatment itself could not induce foci formation (Figure S3E,F).

We also examined whether HSF1 foci formation can be induced by gamitrinib-triphenylphosphonium (GTPP), a well-known mitochondrial HSP90 inhibitor,^43^ because a recent study showed that the HSF1’s nuclear translocation was induced by treatment with GTPP by a fractionation method.^37^ However, we could not observe significant foci formation of HSF1 compared to FCCP-treated cells (Figure S3G,H), which may indicate that the FCCP-induced HSF1 activation might be different from the GTPP-induced proteotoxic stress pathway. All of these results collectively demonstrate that FCCP-induced HSF1 foci formation is independent of ROS, pH changes, and proteotoxic stress. Instead, this can be attributed to mitochondrial thermogenesis.

**Figure 3:**
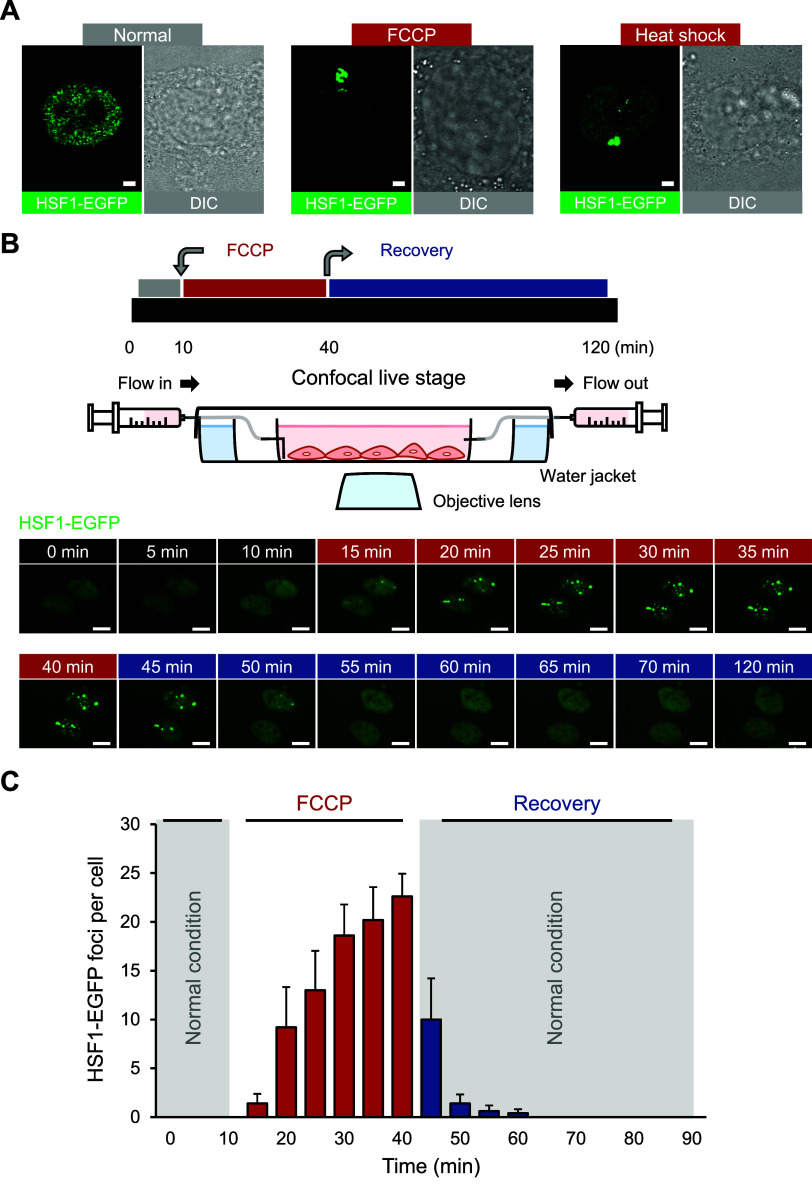
**Real-time imaging of HSF1 foci formation by FCCP treatment.** (**A**) Confocal fluorescent images of HSF1-EGFP foci formation in the stable HSF1-EGFP-expressing cells, under FCCP (100 μM, 1 h) treatment or heat shock (43 °C, 1 h). Scale bar = 2 μm. (**B**) Real-time fluorescence recording of HSF1-EGFP in U2OS HSF1-EGFP-expressing stable cells. The FCCP (100 μM, 30 min) was administered after 10 min of incubation in normal conditions, and recovery was recorded during the incubation in fresh media over the 1 h. (**C**) Time-series graph for the number of counted HSF1-EGFP foci per cell nucleus throughout the FCCP (100 μM, 30 min) treatment and recovery phases for 50 min. Error bars represent the standard error of the mean.

Motivated by the above results, we investigated the potential use of HSF1 foci formation as a real-time recording system that responds to heat conducted from mitochondria. To facilitate the observation of HSF1 foci formation in live cells, we prepared a stable cell line expressing HSF1-EGFP by lentiviral infection (Figure 3A). The HSF1-EGFP foci in the stable cell line was significantly induced by FCCP, and a similar level of foci formation was observed compared to those induced by heat shock at 39 °C (Figure S4A,B) as corresponded to intracellular temperature measured by FDV results. However, HSF1 foci were not formed by FCCP in precooled cells at 32 °C for 2 h, which supports that FCCP-induced foci formation event is a temperature-related process (Figure S4C,D).

Utilizing the GFP fluorescence, we recorded the HSF1-EGFP foci formation at 37 °C immediately following FCCP treatment by real-time confocal microscopy (Figure 3B; supplemental movie 1 and movie 2). In this experiment, we could not observe any HSF1-EGFP foci in cells without FCCP treatment for the first 10 min. However, upon exposure to FCCP, HSF1-EGFP foci emerged immediately and disappeared rapidly when replaced with FCCP-free growth medium (Figure 3B,C). The time-course measurement of the HSF1-EGFP foci number exhibited a strong positive correlation with the presence or absence of FCCP in the cell growth medium (Figure 3C). This result also presented that the HSF1 foci can be induced in a reversible manner by transient heat generation. Consistently, HSF1-EGFP foci formation was also successfully promoted by other mitochondrial protonophores such as BAM15 and CCCP (carbonyl cyanide 3-chlorophenylhydrazone)^25^ (Figure S5A–D). Nuclear HSF1 foci formation following FCCP treatment was again detected to a comparable degree in various other cell lines, including U2OS, HEK293T, MCF10A, and A549 (Figure S5E–H; supplemental movies 3, 4, 5, 6, 7, 8, 9, 10). These observations suggest that HSF1 activation via mitochondrial thermogenesis is conserved in mammalian cells.

### Exothermic Oxygen Reduction Reaction Drives the HSF1 Foci Formation

**Figure 4:**
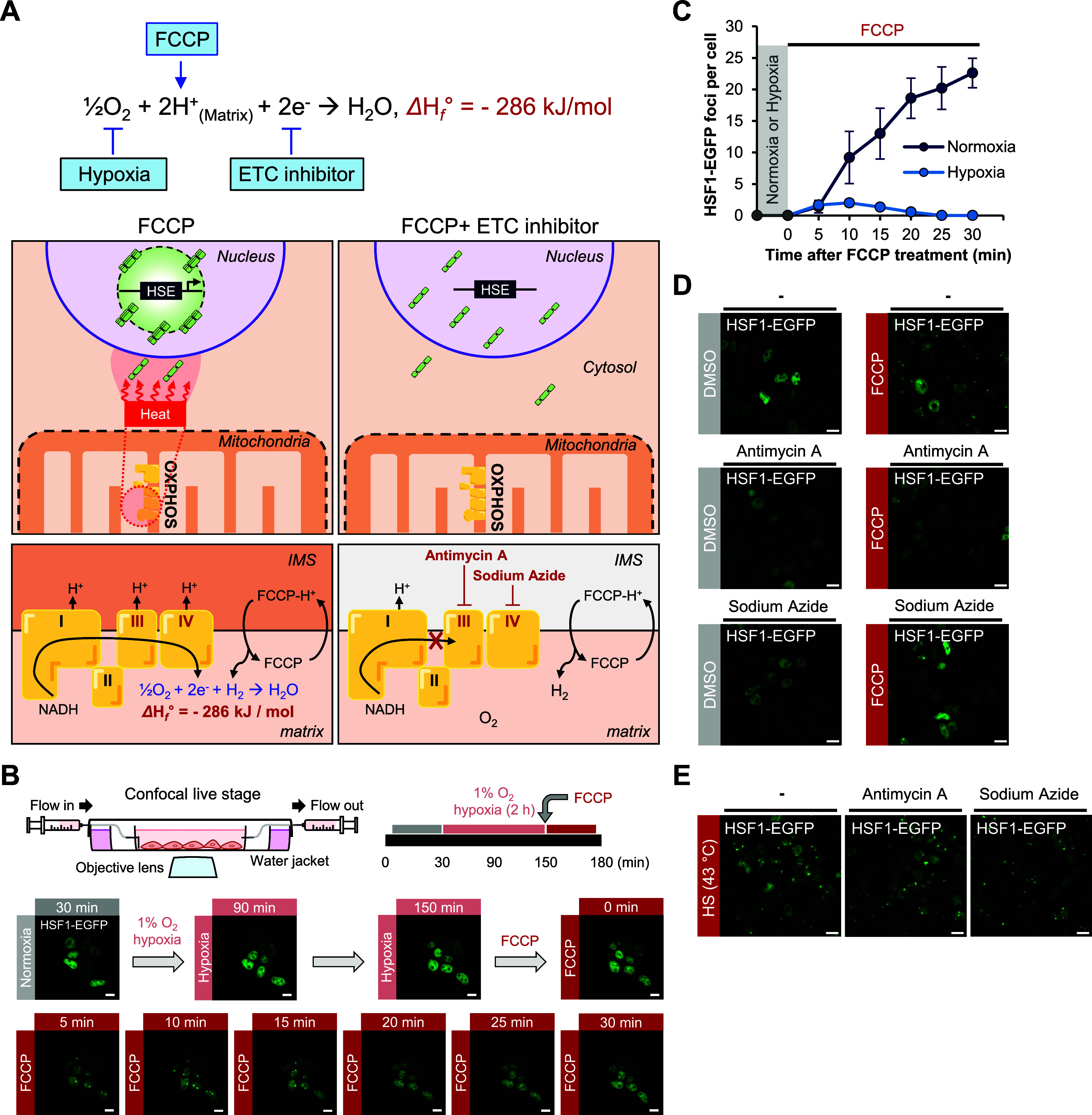
**Mitochondrial oxygen concentration and OXPHOS complex activity are crucial for mitochondrial thermogenesis and HSF1 activation.** (**A**) Schematic view of exothermic mitochondrial oxygen consumption reaction under the FCCP treatment. (**B**) Real-time confocal imaging of HSF1-EGFP stably expressed in HEK293T cells under normoxic (20% O_2_ concentration, 30 min) and hypoxic (1% O_2_ concentration, 2 h 30 min) conditions, with FCCP (100 μM) cotreatment at last 30 min. Scale bar 10 μm. (**C**) The number of counted HSF1-EGFP foci per cell under incubation conditions of 100 μM FCCP and either 1% O_2_ concentration (light blue dots) or 20% O_2_ concentration (dark blue dots). The gray dots represent foci per cell under hypoxia or normoxia without any chemical treatment. Error bars represent the standard error of the mean. (**D**) Confocal images of HSF1-EGFP activation in HEK293T HSF1-EGFP stably expressed cells treated with FCCP (100 μM, 1 h), antimycin A (20 μM, 1 h), and sodium azide (32 μM, 1 h). Scale bar 10 μm. (**E**) Confocal images of HSF1-EGFP activation in HEK293T HSF1-EGFP stably expressed cells treated with antimycin A (20 μM, 1 h) and sodium azide (32 μM, 1 h), under heat shock (43 °C, 1 h) conditions. Scale bar 10 μm.

Mitochondrial thermogenesis, induced by exothermic ORR (1/2O_2_ + 2H^+^ + 2e^–^ → H_2_O),^23^ relies on the abundant availability of reactants (H^+^, O_2_, and electrons) within the mitochondria. Consequently, protonophore-induced mitochondrial thermogenesis is contingent upon a well-maintained oxygen supply and efficient electron transport in the mitochondria. Thus, we hypothesized that either low oxygen levels or impaired electron transport during FCCP treatment may weaken heat generation thereby attenuating formation of HSF1 foci (Figure 4A).

To test this hypothesis, we reduced the oxygen supply to decrease the availability of oxygen molecules in the media and the intracellular environment (Figure 4B; supplemental movie 11). The HSF1-EGFP expressing cells were subjected to hypoxic conditions for 2 h (1% O_2_) prior to incubation with 100 μM FCCP. Using a real-time confocal microscope, we could not detect HSF1 foci formation under prolonged hypoxia of over 2 h (Figure 4C; gray dots), indicating that 2–3 h of hypoxic stress does not activate the HSF1. We observed only 1–2 HSF1 foci per cell within 10 min of FCCP treatment; however, these foci subsequently disappeared in the span of 30 min (Figure 4C; light blue dots). In contrast, under normoxic baseline conditions (20% O_2_, Figure 3C), approximately 10 foci per cell formed within 10 min following FCCP treatment (Figure 4C; dark blue dots). This finding highlights that oxygen supplementation is a prerequisite for HSF1 activation by mitochondrial thermogenesis.

Next, to determine whether HSF1 foci formation is affected by preventing electron supply to the exothermic ORR reaction, cells stably expressing HSF1-EGFP were treated with antimycin A or sodium azide, which inhibit electron flow in the Oxidative Phosphorylation (OXPHOS) complexes III and IV, respectively (Figure 4A). As a result, we observed that HSF1 foci formation was highly reduced upon coincubation of cells with OXPHOS inhibitors and FCCP (Figure 4D). Conversely, HSF1-EGFP foci were distinctly formed in the control group after external heat shock at 43 °C, regardless of treatment with either of the OXPHOS inhibitors (Figure 4E). This result confirmed that OXPHOS inhibitors specifically blocked the mitochondrial thermal activation process, not disturbing the HSF1 system. Overall, here we confirmed that every component (i.e., proton, oxygen, and electrons) in the exothermic mitochondrial ORR is required for generating sufficient mitochondrial heat that can activate HSF1.

### Mitochondrial Thermogenesis Activates HSF1-Dependent Transcriptional Programs

Since heat-induced phosphorylations are prerequisites for the transcriptional active mode of HSF1 in the nucleus,^44−46^ we evaluated whether mitochondrial thermogenesis induced by FCCP treatment induces phosphorylation on HSF1. As shown in the HSF1 Western blot results (Figure S6A), HSF1 molecules moved more slowly in the gel when treated with FCCP, similar to when the heat shock is induced externally.^44^ In contrast, relative to the control group, HSF1 showed no different in-gel migration under intracellular ROS-generating conditions induced by either menadione or rotenone treatment (Figure S6A–D). To clarify whether this slowly migrated HSF1 by FCCP is the phosphorylated product, we treated lambda protein phosphatase (λPPase) in the cell lysates that can actively cleave the phosphorylated groups of HSF1.^47^ Then, we found that the slow migration of HSF1 in both the FCCP or heat shock treated samples were recovered to the normal level by λPPase (Figure S6B). Moreover, we used an antibody specific to phosphorylated HSF1 at serine-326 for direct detection, as this residue is one of the phosphorylation sites triggered by heat shock.^47^ Subsequently, we observed a phosphorylated HSF1 band under both FCCP and heat shock conditions. In contrast, there was no signal in the control, menadione-, and λPPase-treated samples (Figure S6C). These results support the idea that phosphorylation occurs on HSF1 during FCCP-dependent mitochondrial thermogenesis. Taken together, our results suggest that mitochondrial thermogenesis by FCCP may facilitate the transcriptional activity of HSF1 in the nucleus through a similar mechanism by the heat applied externally.

**Figure 5:**
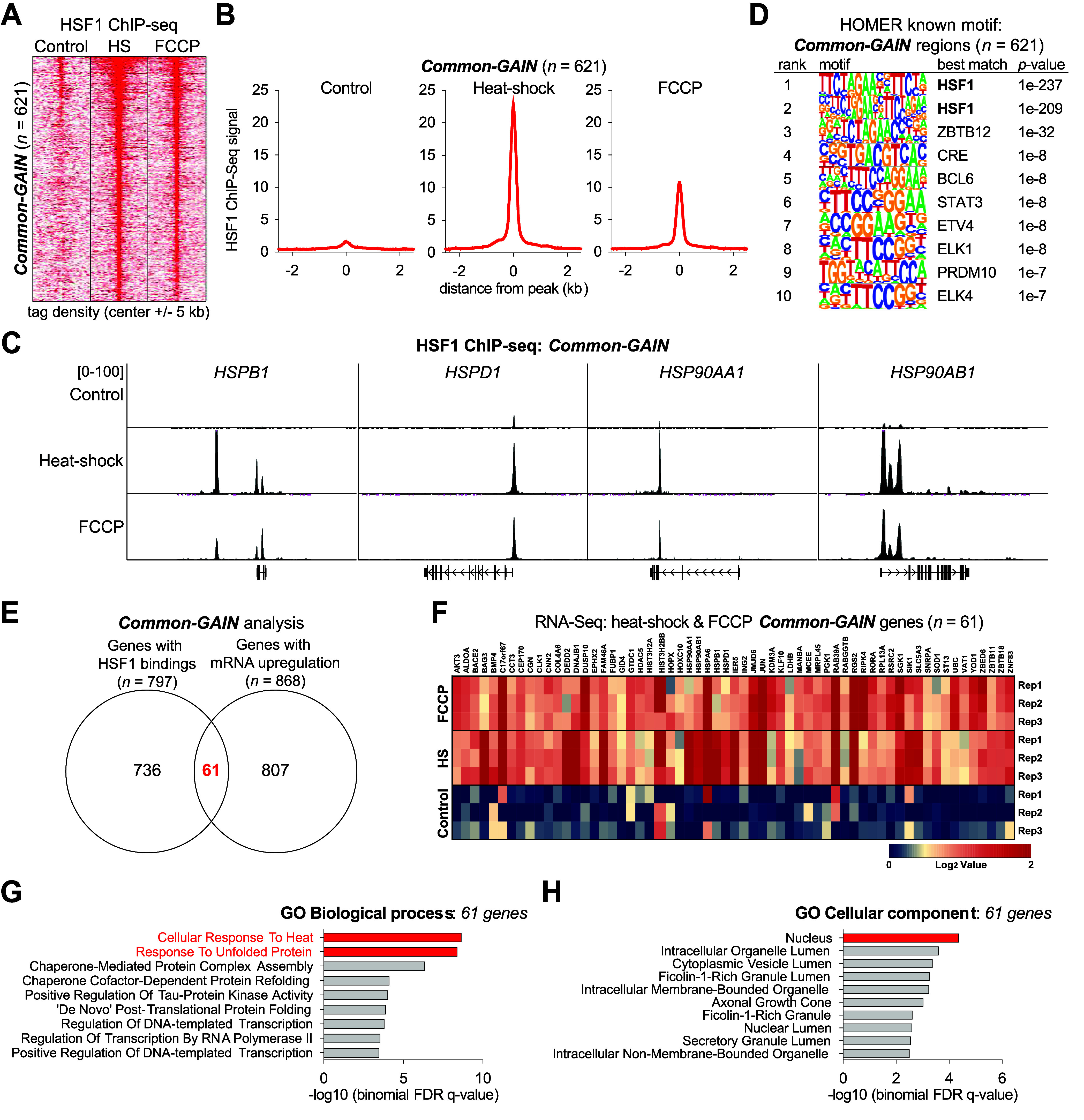
**Chip-seq and RNA-seq analysis for common-GAIN regions of HSF1 expression under heat shock and FCCP treatment**. (**A**) Density plots of HSF1 ChIP-seq signal enrichment at a 5-kb region around the center of heat shock/FCCP cobound regions. Each row represents a single region (*n* = 621). The conditions for heat shock were 43 °C for 1 h, and for the FCCP treatment 100 μM, for 1 h. (**B**) Analysis of ChIP-seq signals on overactive promoters and enhancers under control, heat shock, and FCCP-treatment conditions. The scale of all graphs is calculated based on the distance of the peak (kb). (**C**) Representative HSF1 ChIP-seq profiles at the *HSPB1*, *HSPD1*, *HSP90AA1*, and *HSP90AB1* loci. (**D**) Motif analysis (HOMER) based on HSF1 ChIP-seq results showing enriched motifs in Common-GAIN peaks. Only the top 10 motifs ranked by statistical significance are shown in the logo plot. (**E**) Venn diagram depicting the number of identified HSF1-bound genes (*n* = 797) and mRNA-upregulated genes (*n* = 868), following heat shock or treatment with FCCP. Between the two clusters, there was a total of 61 (*n* = 61) overlapping genes, identified as both being associated with HSF1 and upregulated. (**F**) Heatmap representation of changes in gene expression of heat shock and FCCP Common-GAIN genes (*n* = 61). (**G**, **H**) Gene Ontology (GO) analysis for the identified 61 heat shock/FCCP cobound region genes of (**G**) the associated biological processes and (**H**) the subcellular compartment (Cellular Component).

To further validate this hypothesis, we investigated whether nucleus-localized HSF1 accumulation drives the expression of heat shock response-related programs. Then, we mapped the genome-wide binding of HSF1 using chromatin immunoprecipitation sequencing (ChIP-seq) to determine whether HSF1 binds to heat shock response genes. Meta-analysis of the ChIP-seq data sets revealed 621 sites that commonly gained HSF1 signals in the promoter regions, termed Common-GAIN regions (Figure 5A). Moreover, we found sites that recruited HSF1 under either heat shock or FCCP-treatment conditions, termed the HS-GAIN and FCCP-GAIN regions, respectively (Figure S7). Notably, the average intensity of FCCP-mediated HSF1 binding to Common-GAIN sites was reduced to ∼50% of that compared to heat shock-dependent HSF1 activation, corroborating that FCCP treatment increased nuclear temperature to levels lower than 43 °C (Figure 5B,C). This result is in good agreement with our previously measured mean temperature of the entire cellular area (39.6 °C) using FDV under FCCP treatment (Figure S1F). The motif analysis of the Common-GAIN regions returned classical HSF1 binding motifs, although the possibility of engagement of other transcription factors could not be excluded (Figure 5D). We confirmed with the luciferase assay system that the HSPD1/HSPE1 promoter, identified as one of the Common-GAIN regions (Figure 5C), can be activated under both external heat shock and FCCP treatment conditions (Figure S8A,B).

Among the 797 genes in which single or multiple HSF1-binding peaks were found in the promoter regions, RNA-seq analysis further revealed that 61 genes in the Common-GAIN regions have a positive correlative transcription activation pattern between FCCP-treated and external heat shock conditions (Figure 5E,F and Figure S8C). Furthermore, gene ontology (GO) analysis revealed that these 61 genes were significantly associated with the nuclear response to heat shock stress, exemplified by “cellular response to heat” and “response to unfolded protein”, indicating that the overall transcriptional output of the FCCP treatment phenocopies that of the external heat shock stimulus (Figure 5G,H and Figure S8D,E). Taken together, our findings suggest that FCCP-mediated heat generation activates HSF1 to bind to heat shock response genes, leading to increase in the transcriptional level of HSPs similar to external heat shock conditions.

## Discussion

In this work, we used HSF1 as a molecular sensor to monitor how mitochondrial heat affects intracellular biological events. Consequently, we found that HSF1 foci formed in the nucleus when we treated protonophores to initiate the mitochondrial reaction for the heat-releasing oxygen reduction reaction. At the same time, we also observed that HSF1 foci formation is reversible by controlling the oxygen consumption in the mitochondria. Our integrative HSF1 ChIP-seq and RNA-seq experiments verified that FCCP activates the functional HSF1 pathway in a similar manner under external heat stress conditions.

Our results revealed that mitochondrial thermogenesis can modulate the activity of intracellular thermosensing proteins, such as HSF1, in live cells. This suggests that mitochondria-generated heat could act as an intracellular signal that may lead to protein conformational alterations without intermediate molecular interactions. Compared to the targeted signaling by molecular transfer events (i.e., molecular conversion and translocation,^37,48,49^ molecular interactions, or modifications), mitochondrial heat can be a global retrograde signal that spatiotemporally affects numerous proteins. In mammalian cells, many proteins have a low melting temperature around 40 °C,^50^ and proteins with intrinsically disordered proteins (IDRs) can occur liquid–liquid phase separation (LLPS) in the range of 40 °C.^51−55^ Notably, HSF1 possesses a long IDR domain (221–383 aa) that can induce LLPS.^56^ Therefore, it is expected that many of such low-melting-temperature or IDR-containing proteins can be the primary effectors of mitochondrial heat signaling.

It is noteworthy that HSF1 has been studied for its role in mitochondria and nucleus communication. A group of studies has shown that HSF1 can sense mitochondrial misfolding stress and mediated mitochondrial protein unfolded response (UPR^mt^) in the nucleus.^9,37^ Other studies have shown that HSF1 can be activated by mitochondrial ROS and protect cells under the mitochondrial stress conditions.^37,57,58^ Our study showed that mitochondrial generated heat can also activate HSF1. Our data also reinforce the hypothesis that HSF1 is a key protein acting as a master message-transducer between the mitochondria and the nucleus.

We also provided a new chemical interpretation of mitochondrial thermogenesis, based on the exothermic property of the oxygen reduction reaction (1/2O_2_ + 2H^+^ + 2e^–^ → H_2_O), which is well-recognized in other research fields.^23,24,59^ To the best of our knowledge, our study first connects the exothermic enthalpy change of this oxygen consumption reaction (Δ*H*_f_° = −286 kJ/mol) to the mitochondrial thermogenesis and suggests that this is likely the primary thermogenic reaction in mitochondria. We validated that every component (i.e., protons, oxygen, and electrons) in this reaction is crucial for sufficient mitochondrial thermogenesis and the subsequent upregulation of HSF1. As the oxygen consumption rate is regarded as a reliable indirect measurement standard for thermogenic events in the metabolism research field,^60^ we believe that our theorem may be accepted in the field.

We also anticipate that our ORR-driven thermogenesis model can establish a new connection between mitochondrial respiration and mitochondrial thermogenesis, potentially in all actively respiring cells. There is accumulated evidence suggesting that not only brown adipose tissue but also various tissue-originated mammalian cells, including cancer cells, exhibit active mitochondrial respiration activities.^19,61−64^ Therefore, we expect that these actively respiring cells may experience an increased intracellular temperature and could activate the HSF1 signaling pathway, possibly due to active ORR-driven mitochondrial thermogenesis. Since HSF1 foci formations have been observed in several aggressive human tumors,^8,30^ it would be intriguing to further investigate this mito-ORR and HSF1 axis or mitochondrial heat signaling in cancer research.

In summary, our current study demonstrated that mitochondrial-generated heat is sufficient to activate HSF1 independent of pH, ROS, and proteotoxic stress. It provides supporting evidence to consider intracellularly generated heat as a distinct signal that promotes subsequent changes in cell homeostasis. Our work may serve as a basis for future investigations to delineate the complex relationships between various mitochondrial thermogenic events and the activation of heat-sensitive proteins under diverse physiological and pathological contexts.

## Supporting Information Available

Materials and methods, plasmid information, additional figures, movie legends, references (PDF)
Supplementary Dataset 1. 61 genes associated with Figure 5F. Raw data (XLSX)
Transparent Peer Review report available (PDF)
Movie 1. Live imaging of U2OS cells stably expressing HSF1-EGFP shown in the Figure 3B, 0−10 min: normal growth media, 10−40 min: FCCP-treated media, 40−120 min: normal growth media (AVI)
Movie 2. Wide view of live cell imaging in Figure 3B (AVI)
Movie 3. Live imaging of MCF10A cells stably expressing HSF1-EGFP shown in the Figure S5E, 0−30 min: normal growth media, 30−82 min: FCCP-treated media (AVI)
Movie 4. Wide view of live cell imaging in Figure S5E (AVI)
Movie 5. Live imaging of HEK293T cells stably expressing HSF1-EGFP in normal growth media for 30 min shown in Figure S5F (AVI)
Movie 6. (Continued from Movie 5) Live imaging of HEK293T cells stably expressing HSF1-EGFP in FCCP-treated media for 30 min shown in Figure S5F (AVI)
Movie 7. (Continued from Movie 6) Live imaging of HEK293T cells stably expressing HSF1-EGFP in normal growth media for 30 min shown in Figure S5F (AVI)
Movie 8. Live imaging of U2OS cells stably expressing HSF1-EGFP shown in Figure S5G, 0−30 min: normal growth media, 30−67 min: FCCP-treated media (AVI)
Movie 9. Wide view of live cell imaging in Figure S5G (AVI)
Movie 10. Live imaging of A549 cells stably expressing HSF1-EGFP shown in Figure S5H, 0−30 min: normal growth media, 30−35 min: FCCP-treated media, 35−67 min: normal growth media (AVI)
Movie 11. Live imaging of HEK293T cells stably expressing HSF1-EGFP after FCCP treatment under hypoxia condition (1% O_2_) shown in Figure 4B (AVI)

## Supplementary Material

oc3c01589_si_003.pdf

oc3c01589_si_004.avi

oc3c01589_si_005.avi

oc3c01589_si_006.avi

oc3c01589_si_007.avi

oc3c01589_si_008.avi

oc3c01589_si_009.avi

oc3c01589_si_010.avi

oc3c01589_si_011.avi

oc3c01589_si_012.avi

oc3c01589_si_013.avi

oc3c01589_si_014.avi
