# Supplementary material for: Mitochondrial Thermogenesis Can Trigger Heat Shock Response in the Nucleus
Source: ACS Cent Sci. 2024 Jun 3;10(6):1231–41. doi: 10.1021/acscentsci.3c01589 (PMC11212142; doi:10.1021/acscentsci.3c01589)
Supplement: Supplementary file 1 — oc3c01589_si_001.pdf [file oc3c01589_si_001.pdf]

Supporting Information

Mitochondrial Thermogenesis Can Trigger Heat Shock Response in the Nucleus

Myeong-Gyun Kang[1,†], Hwa-Ryeon Kim[2,†], Hee Yong Lee[1,†], Chulhwan Kwak[1,5], Hyewon Koh[1], Byoung Heon Kang[3], Jae-Seok Roe[2,*], Hyun-Woo Rhee[1,4,*]

[1]Department of Chemistry, Seoul National University, Seoul 08826, Korea

[2]Department of Biochemistry, Yonsei University, Seoul 03722, Korea

[3]Department of Biological Sciences, Ulsan National Institute of Science and Technology (UNIST), Republic of Korea

[4]School of Biological Sciences, Seoul National University, Seoul 08826, Korea

[5]Current address: Department of Neurosurgery, Stanford University, Stanford, CA, 94305, USA

*To whom correspondence should be addressed: rheehw@snu.ac.kr and jroe@yonsei.ac.kr

†These authors contributed equally

Table of contents:

Materials and methods S2-8

Plasmid information S9

Figure S1 .... S10

Figure S2 .... S11

Figure S3 .... S12

Figure S4 .... S13

Figure S5 .... S14

Figure S6 .... S15

Figure S7 .... S16

Figure S8 .... S17-18

Supplemental Movie legends S19

Reference S20

Materials and Methods

Plasmids and Cloning. Genes were cloned into appropriate vectors using standard enzymatic restriction digestion and ligated with the T4 DNA ligase. The PCR products were digested with the same restriction enzymes and ligated into cut vectors (e.g., pcDNA5 and pFUGW). For pcDNA5, the CMV promoter was used, whereas for pFUGW, the UBC promoter was used for expression in mammalian cells. Plasmid information depicts the gene constructs used in this study.

Cell Culture and Transfection. The HEK293T cells were obtained from ATCC (Manassas, VA, USA), while the U2OS and A549 cells were obtained from the Korean Cell Line Bank. All cell lines were cultured according to standard mammalian cell culture protocols at 37 °C under 5% CO_2 in a humidified incubator. The MCF10A cell line (ATCC-CRL-10317) was a gift from Mi-Ock Lee (Seoul National University) and cultured in DMEM / F12 (1:1) (Thermo Fisher Scientific, Cat # 11330-032) supplemented with 5% FBS, 20 μg/ml EGF (Peprotech, 1 mg), 0.5 mg/ml hydrocortisone (Sigma Aldrich, Cat # H-0888-1g), and 10 μg/ml insulin (Sigma Aldrich, Cat # I9278-5ML). Cell lines at 60–80% confluence was transiently transfected using polyethyleneimine (PEI, Polysciences, Cat # 23966).

Preparation of Stable Cell Lines. In this study, stable cell lines were prepared via lentiviral infection. For the generation of lentivirus, the HEK293T cells were cultured in DMEM (Corning, Cat # 10-013-CV) supplemented with 10% FBS and 100 U/ml Penicillin-Streptomycin (Thermo Fisher Scientific, Cat # 15140163) at 37 °C under 5% CO_2. The cells were grown in a 6-well cell culture plate containing 2 ml of medium. Lentivirus were generated by co-transfection of 60–80% confluent cells using PEI and vector plasmids. The transfection per well was performed by addition of a mixture consisting of 1000 ng transfer vector (pFUGW), 375 ng of pMDL.g/pRRE (Addgene, #12251), 375 ng of pRSV-REV(Addgene, #12253) or 250 ng of pMD2.g (a gift from Jiyoung Park in UNIST), premixed with 4 μg of PEI in 200 μL DMEM without FBS for 10 min. Following 2–3 h incubation, the medium was replaced with complete DMEM to allow the production of the recombinant lentivirus in transfected cells. Then, medium was collected 24 h after transfection and cells were incubated with fresh complete DMEM once more for additional virus production. Each collected virus-containing medium was filtered through syringe-driven filters (JetBiofill, Cat # J1.F404.013N). Cell lines (HEK293T, U2OS, A549, MCF10A) were cultured in a 6-well cell plate under appropriate conditions and when at 60% confluence, the medium was replaced with the filtered medium containing virus. For lentiviral infection, MCF10A cells were incubated for 3 h and other cell lines were incubated for 16 h. Subsequently, the cells were cultured in fresh complete media and scaled up to the desired experimental volume.

Reagents for various stress conditions. Carbonyl cyanide-p-trifluoromethoxyphenylhydrazone (FCCP, Sigma Aldrich, Cat # C2920), carbonyl cyanide m-chlorophenyl hydrazone (CCCP, Sigma Aldrich, Cat # C2759), and BAM15 (Sigma Aldrich, Cat # SML1760) were used for inducing mitochondrial uncoupling process. Menadione (Sigma Aldrich, Cat # M5625) and N-acetyl-L-cysteine (NAC, Sigma Aldrich, Cat #

A9165) were employed as reactive oxygen species (ROS) generator and quencher, respectively. Rotenone (Sigma Aldrich, Cat # R8875), antimycin A (Sigma Aldrich, Cat # A8674), and sodium azide (Sigma Aldrich, Cat # S2002) were utilized as electron transport chain inhibitors. To induce proteotoxic stress, Gamitrinib-triphenylphosphonium (GTPP, a gift from Byoung Heon Kang in UNIST) was employed. Cycloheximide (Alfa Aesar, Cat # J66901) and puromycin (InvivoGen, Cat # ant-pr-1) were used to block protein synthesis.

Confocal Imaging and Analysis. To visualize the subcellular localization of the endogenous or overexpressed proteins, cells were seeded on coverslips (MARIENFELD, Cat # HSU-0111580). For fixed cell imaging, cells were fixed with 4% paraformaldehyde and washed with phosphate-buffered saline (PBS) twice. Cells were permeabilized by 0.1% Triton X-100 (Sigma Aldrich, Cat # 9536-19-5) in PBS for 5–10 min at room temperature. After washing with PBS twice, cells were incubated in a blocking solution (2% BSA in PBS) for 1 h. Immunolabeling was conducted by sequentially adding primary antibodies followed by secondary antibodies. Cells were incubated were anti-HSF1 (1:500 dilution, CST, Cat # 4356) or anti-Histone 3 (1:3000 dilution, Santa Cruz, Cat # sc-517576) in a blocking solution overnight at 4 °C. After washing with PBS three times, cells were incubated with Alexa Fluor-conjugated anti-mouse 647 (1:2000 dilution, Invitrogen, Cat # A-21235) or anti-rabbit 488 (1:2000 dilution, Invitrogen, Cat # A-11008) for 1 h at room temperature, followed by washing with PBS three times. Immunofluorescence images were obtained and analyzed using an SP8 X Leica microscope (NICEM, Seoul National University, Seoul, Republic of Korea) with an objective lens (HC PL APO 100x/1.40 OIL), a white light laser (470–670 nm, 1 nm tunable laser), and a HyD detector, which was controlled using the LAS X software and FV3000 microscope (Oylmpus) with an objective lens (UPLXAPO 100XO), which was controlled using the Olympus Fluoview software. For HSF1 imaging experiments, HSF1 foci were counted manually and statistically quantified using GraphPad Prism 10 software.

Flow Cytometry Analysis for Mitochondrial Membrane Potential and Temperature Measurement by ETAC. Cells were seeded in 6-well plates for flow cytometry analysis. The next day, cells were incubated for 30 min with 200 nM TMRE (Sigma Aldrich, Cat # 115532-52-0) or 0.5 μM ETAC (a gift from Young-Tae Chang)[1]. Cells were then washed three times with the medium and incubated overnight in fresh medium. The cells stained by each dye were further incubated with either 100 μM FCCP for 1 h and 30 μM menadione for 30 min. A steady state indicates the absence of chemical treatment. For the following flow cytometry analysis, cell suspensions were prepared by trypsin-EDTA (0.25%)-based cell detachment and diluted fivefold with fresh medium. Samples (10000 cells/events) were examined using a FACSCanto II (BD Bioscience) or flow cytometry LX (Beckman Coulter) according to their fluorescence signals. All flow cytometry data were processed using the FlowJo 10 or CytExpert software.

Intracellular Temperature Sensing by Cellular Thermoprobe 'FDV'. To use FDV (Funakoshi, Cat# FDV-0005)[2], 1 mg powder of FDV was reconstituted in 100 μl of 5% glucose in ultrapure water to prepare a 1% w/v stock solution freshly before each experiment. The stock solution was incubated at 4 °C at least overnight with protection from light to obtain full extension of the FDV. For the calibration curve, Cells were cultured on 96-well black plates (SPL Life Sciences, Cat # 30296) using the same conditions as described in "Cell

Culture" section. The media volume was 200 µl for each well. To measure the fluorescence, we used cell cultures at 90 % confluence and cells were washed with 5% glucose. Then, cells were treated with 0.05% of FDV in 5% glucose for 10-20 minutes followed by washing with Dulbecco's phosphate-buffered saline (DPBS, Thermo Fisher Scientific, Cat # 21300025) three times. After checking FDV staining in cells by epi-microscope, the fluorescence was measured using a Synergy ™ H1 microplate reader (BioTek Instruments, Inc.). A wavelength of 458 or 473 nm was used for excitation. The emission range was 490-530 nm and 570-610 nm. The temperature was initially set at 30 °C and then sequentially raised by 5 °C. Before each measurement, there was a wait time for 15 min to allow the plates to heat adequately. Measurements were taken quintuplicate, and the median value was used for the analysis. The experiment ended at a final temperature of 45 °C. For the FCCP treatments, cells were used at 90 % confluence, and the initial medium was replaced with an equal volume of FCCP diluted in culture media. After FDV staining in the same way with the calibration method, the fluorescence was measured on a microplate reader set at a temperature of 37 °C. For data analysis, the ratio of FDV and the temperature change were estimated from the formula of the fitted standard curve.

pHluorin2 Fluorescence Measurement. Excitation scans were performed using a Synergy ™ H1 microplate reader (BioTek Instruments, Inc.). HEK293T cells were plated on 96-well black plates (SPL Life Sciences, Cat # 30296) and transiently transfected with 1000 ng pHluorin2 plasmid DNA mixed with 4 µg of PEI (Polysciences, Cat # 23966). After 24 hours of transfection, cells were washed with DPBS. Plates were placed into the microplate reader for a 10 min incubation at 37°C and measured. A wavelength of 520 nm was used for emission. The excitation was scanned between 300 nm and 490 nm with a 2 nm step. Measurements were taken from triplicate samples, and the median value was used for the analysis. For the pH standard curve, the calibration buffer contained the following: 50 mM MES buffer (pH 5.5, 6.0, and 6.5), 50 mM Tris · HCl (pH 7.0, 7.5, and 8.0).

Live Cell Imaging and Analysis. For live cell imaging, the cells were cultured in a 30 mm glass bottom confocal dish (SPL Life Sciences, Cat # 100350) or 18mm glass (MARIENFELD, Cat # HSU-0111580) coated with poly-L-lysine solution (Sigma, Cat # P8920-100ML). Using an ExFluorer live cell imager (Live Cell Instrument, Namyangju, Republic of Korea). For HSF1-EGFP, the FITC channel (488 nm) was used. The confocal dishes containing the cultured cells were fastened tightly without a dish cover and placed on a stage-on incubator, maintained at 37 °C under 5% CO_2 in a humidified environment. For imaging the HSF1-EGFP, exposure time was set as 200 ms. The initial volume of the medium was 2 ml. For the FCCP treatment, 1 ml of medium was carefully removed from the cell culture dish, subsequently 1 ml of 2×FCCP-containing medium was added. For cell recovery, 1.5 ml of medium in the cell culture dish was replaced with an equal volume of fresh culture medium, before subsequently replacing the entire total volume of medium in the cell culture with new fresh culture medium.

Live Cell Imaging for FCCP Treatment-Recovery with or without Hypoxia. To conduct real-time confocal imaging of HSF1-EGFP in various cell lines, cells were cultured using the same methods with 'Live

Cell Imaging and Analysis'. A 'Incubator T Series' (Live Cell Instrument, Namyangju, Republic of Korea) is on the FV3000 microscope (Oylmpus) stage, which precisely controls the gas, temperature, and humidity of the stage incubator. For imaging, all cell lines were cultured according to standard mammalian culture protocols at 37 °C and 5% CO_2 in a humidified incubator. Cell lines at 70-90 % confluence moved the cells to the confocal live stage. Live stage maintained the same condition as the cell culture incubator. The media were replaced through the flow out/in tube by syringe. Images were obtained using the same methods with 'Confocal Imaging and Analysis'. For hypoxia condition, the live stage incubator O_2 concentration was regulated from 20% to 1%. Cells were in the 1% hypoxia condition at least 2 h for fully consumed the media and intracellular oxygen.

Western Blotting. For all cell lines used in this work, the cells were lysed under the appropriate conditions per experiment (such as FCCP treatment or 43 °C heat shock). Cells were lysed in RIPA lysis buffer (Elpis Biotech, Cat # EBA-1149) or PBS buffer containing 2% SDS (Sigma Aldrich, Cat # 436143) and 1× protease inhibitor cocktail (Invitrogen, Cat # 78438), for 10-20 min at 4 °C. In the case of lysis with 2% SDS solution, lysates were transferred to 1.5 ml tubes and cleared by ultrasonication (QSONICA, Q800R3) for 6 min in a cold-water bath. Lysates were then centrifuged at 15,000 × *g* for 10 min at 4 °C, and the supernatants were collected. Lysate samples were boiled at 95 °C for 5 min after mixing with the SDS-PAGE loading buffer. Subsequently, the proteins were loaded on bis-tris gels for SDS–PAGE (Invitrogen) and transferred to the nitrocellulose membrane (PALL, 0.2 μm). After ponceau staining (1 min in 0.1% (w/v) Ponceau S in 5% acetic acid/water) and blocking with 2% skim milk solution in 0.1% Tween-20 in 1× Tris-buffered saline (1× TBST), the membrane was subjected to immunoblotting with the indicated antibodies. The primary antibodies used for western blotting were anti-HSF1 (1:500 dilution, CST, Cat # 4356), anti-HSF1 phospho S326 (1:1000 dilution, abcam, Cat # ab115702), anti-Histone 3 (1:3000 dilution, Santa Cruz, Cat # sc-517576), anti-V5 tag antibody (1:5000 dilution, Invitrogen, Cat# R960-25) and anti-GAPDH (1:2000 dilution, Santa Cruz, Cat # sc-32233). Secondary antibodies, including anti-mouse (1:3000 dilution, CellNest, Cat # CNG004-0005) and rabbit-horseradish peroxidase (1:3000 dilution, CST, Cat # 7074S) were used for blotting. The membrane was incubated with primary antibody diluted in blocking solution for 1-2 h followed by washing 3 × 5 min with 1× TBST. The membrane was then incubated with a secondary antibody solution for 30-60 min. Membrane development was performed using the Clarity reagent (Bio-Rad) and imaging on a gel doc machine (cytiva, Amersham™ ImageQuant™ 800) after washing 3 × 5 min with 1× TBST.

APEX2 reaction with Endogenously generated ROS. HEK293T cells were cultured in the same condition as described in "Cell Culture". After 24 h of transfection, 250 μM DBP in DMEM was treated to the APEX2-expressed cells for 30 min, and then, 30 μM menadione was added for an appropriate time. The cells were washed with DPBS containing 5 mM Trolox (Sigma Aldrich, Cat # 238813), 10 mM sodium azide, and 10 mM sodium ascorbate (Sigma Aldrich, Cat # A4034) three times, and RIPA lysis buffer was added after removing DPBS. The cells were lysed for 10 min at 4 °C, and the sample was loaded onto a 10% sodium dodecyl sulfate-polyacrylamide gel electrophoresis gel for separation at 220-250 V for 60 min. After separation, proteins on the gel were transferred to a nitrocellulose membrane at 25-30 V for 180 min. The protein loading level was checked by Ponceau staining; the stain was removed by washing with 1× TBST.

Membrane blocking was performed with a solution of 2% (w/v) dialyzed BSA in 1× TBST at 4 °C overnight, or at room temperature for 1 h. The blots were immersed in streptavidin-horseradish peroxidase in blocking buffer (1:10,000 dilution, Thermo Fisher Scientific, Cat # 21126) at room temperature for 30 min and then rinsed with blot blocking buffer three times for 5 min, before development using the Clarity reagent (Bio-Rad) and imaging on a gel doc machine (cytiva, Amersham™ ImageQuant™ 800). For assessing comparative enzyme expression levels, the membrane was stripped with a stripping buffer (100 mM 2-mercaptoethanol, 2% SDS, 62.5 mM Tris-HCl, pH 6.8). Blots were washed three times, 5 min per wash, in the 1× TBST buffer and incubated in a 5% blocking buffer for 1–2 h. They were then incubated in 10 mL of anti-V5 tag antibody solution diluted in the blocking buffer for 1–2 h at room temperature. Blots were subsequently washed 3 × 5 min in 1× TBST prior to incubation with 10 mL of anti-mouse-HRP diluted in the blocking buffer for 30–60 min at room temperature. The membranes were then washed again for three times, 5 min per wash in 1× TBST buffer before developing using the Clarity reagent (Bio-Rad), as described above.

Dephosphorylation of proteins with lambda protein phosphatase (λPPase). The MCF10A cells were lysed using RIPA lysis solution containing 1× protease inhibitor cocktail after being subjected to appropriate experimental conditions (such as FCCP treatment or 43 °C heat shock). Lysates were then centrifuged at 15,000 × *g* for 10 min at 4 °C, and the supernatants were collected in the new 1.5 ml tubes. NEBuffer Pack for Protein MetalloPhosphatases, $MnCl_2$ and 400 units of λPPase (New England Biolabs, Cat # P0753S) were sequentially added into supernatants to dephosphorylate the proteins, following the manufacturer's instructions. Mixture was incubated at 30 °C for 30 min, and the proteins were loaded on bis-tris gels for SDS–PAGE (Invitrogen). Immunoblotting with anti-HSF1, histone H3, and GAPDH was conducted as described in the "Western Blotting" section.

Luciferase assay. HEK293T cells were cultured on 96-well plate (Corning, Cat # 354620) in the same condition as described in "Cell Culture". After 24 h of transfection using 1:1 mixture of a firefly and *Renilla* luciferase plasmids, cells were subjected to a stressed condition for 1 h followed by washing with DPBS three times.

Reagent was prepared (Dual-Glo® Luciferase Assay System, Promega, Cat # E2920) as below.

- Luciferase Reagent: Luciferase Buffer was transferred to Luciferase Substrate in the bottle and store at -70 °C (maximum of 6 months).
- Stop & Glo (S&G) Reagent: 50× S&G substrate was diluted with S&G buffer to prepare 1× concentration in the amber bottle and vortexed for 10 sec. It was prepared immediately for every experiment.

The DPBS in the plate was replaced with Luciferase Reagent in an equal volume to the culture medium, subsequently incubated for 10 min. Then, firefly luciferase activity was measured using SpectraMax i3x (Molecular Devices). After measurement, S&G Reagent was added to the plate, followed by incubation for 10 min. *Renilla* luciferase activity was measured in the same plate order as the firefly luciferase activity measured.

Chromatin immunoprecipitation (ChIP). ChIP experiments were performed as previously described[3]. Briefly, a concentration of 3×10^7 single trypsinized HEK293T cells was used. The trypsinized cells were crosslinked with 1% formaldehyde, followed by quenching in 0.125 M glycine, and washed with PBS. The cell pellets were lysed with 1200 µl of cell lysis buffer [10 mM Tris-Cl pH 8.0, 10 mM NaCl, 0.2% NP-40] supplemented with protease inhibitor cocktail (Roche, Cat # 11697498001) and 1 mM DTT. Chromatin was isolated by centrifugation at 7,400 rpm for 30 s. The pellet was gently resuspended in a 1200 µl of nuclei lysis buffer [50 mM Tris-Cl pH 8.0, 10 mM EDTA, 1% SDS] containing protease inhibitor and 1 mM DTT. The chromatin lysate was sonicated (Diagenode, Bioruptor pico) for 10 cycles (30 s, and 30 s off), and the sonicated chromatin mixture was then incubated for 1 h with 60 µg of rabbit IgG, and 60 µl of Protein A magnetic beads (Invitrogen, Cat # 10001) for pre-clearing. Immunoprecipitation was conducted with 6 ml of pre-cleared chromatin, 30 µl of the HSF1 antibody (CST, Cat # 4356), and 60 µl of Protein A magnetic beads overnight at a 4 °C rotator. The following day, the immune complexes were washed with IP Wash I Buffer, twice with high-salt buffer, once with IP Wash II buffer, and finally twice with TE (pH 8.0). The washed immune complexes were eluted by incubation with 200 µl of elution buffer [1% SDS and 0.1 M NaHCO3] for 30 min at a 45 °C thermomixer, shaking at 1,000 rpm. The eluate was de-crosslinked with RNase A (1 µg/µl) and 0.25 M NaCl and incubated overnight at a 65 °C water bath. The next day, the eluate samples were incubated for 2 h in a Proteinase K (NEB, Cat # P8107S) solution, and the immunoprecipitated DNA was purified with a QIAquick PCR purification kit (QIAGEN, Cat # 28106;) in 50 µl of EB (elution buffer).

ChIP-seq Library Construction. The ChIP-seq libraries were constructed using 40 µl of purified ChIP DNA and NEXTflex™ ChIP-seq kit (PerkinElmer, Cat # NOVA-5143-02) according to the manufacturer's instructions. Briefly, ChIP DNA was end-repaired and size-selected (250–300 bp) using AMPure XP beads (Beckman, Cat # A63881). All subsequent procedures, from adenylation to PCR amplification, were performed according to the ChIP-seq library construction steps. The quality of the ChIP-seq libraries was determined by a Bioanalyzer using the High Sensitivity chip (Agilent), and the average size of the ChIP-seq libraries ranged from 250 to 350 bp. For multiplexing, equal molar quantities of libraries were combined by considering the sequencing depth per sample (20–40 million reads per library). The ChIP-seq libraries were sequenced using the Illumina NextSeq platform with single-end reads of 76 bases.

RNA-seq Library Construction. Total RNA was extracted using the QIAzol reagent (QIAGEN, Cat # 79306) according to the manufacturer's instructions. RNA-seq libraries were constructed using 5 µg of purified RNA and a NEXTflex™ Rapid Directional mRNA-seq kit (PerkinElmer, Cat # NOVA-5138-11). Briefly, purified RNA was poly-A selected and fragmented using a fragmentation enzyme. After first- and second-strand cDNA synthesis from a fragmented RNA template, the steps from adenylation to PCR amplification were performed according to the RNA-Seq library construction steps.

Data analyses of RNA-seq.

(1) Identification of DEGs. Raw reads from RNA-seq were aligned to the human reference genome assembly (mm9) using STAR mapping tool. The relative transcript abundances were measured in Reads Per Kilobase of transcript per Million mapped reads (RPKM) from Cufflinks.

(2) GSEA analysis. GSEA was performed according to the instructions. The MSigDB v7.0 and reference database were used for transcriptional differences between each treatment.

(3) Gene ontology (GO) analysis of genes. A list of the defined Common-GAIN 61 genes was used as an input for GO analysis with the AmiGO tool.
The RNA-seq results, including Common-GAIN 61 genes, are available in the Supporting Information.

Statistics. The **s**tatistical analyses were performed using excel and GraphPad Prism 10 software. The probability values (P-values) were obtained using an unpaired two-paired T test or one way Analysis of variance (ANOVA). The P-values lower than 0.05 ($P < 0.05$) were considered statistically significant, and the following symbols were used: * ($P < 0.05$), ** ($P < 0.01$), *** ($P < 0.001$), **** ($P < 0.0001$), “ns” indicates not significant ($P > 0.05$).

Plasmids Information

Name (expected size)	Features	Promotor/ Vector	Details
HSF1-linker-EGFP	*BsiWI*-HSF1-*HindIII*-13 a.a linker-*NotI*-EGFP-*STOP-BlpI-EcoRI*	UBC / pFUGW	13 a.a linker: GAPGSAGSAAGSG
MTS-V5-APEX2	*KpnI*-Mito-*BamHI-NheI*-V5-APEX2-*Stop-NotI*	CMV/ pcDNA5	Mitochondrial targeting sequence (MTS): MLATRVFSLVGKRAISTSVCVRAH[4]
SCO1-V5-APEX2	*KpnI*-SCO1-V5-APEX2-*Stop-NotI*	CMV/ pcDNA5	SCO1: Inner mitochondrial membrane (IMM) protein
TDRKH-V5-APEX2	*HindIII-KpnI*-TDRKH-*BamHI-NheI*-V5-APEX2-*STOP-NotI-XhoI*	CMV/ pcDNA5	TDRKH: Outer mitochondrial membrane (OMM) protein
GBP-V5-APEX2	*AflII-HindIII-KpnI*-GBP-*BamHI*-V5-APEX2-*STOP-NotI-XhoI*	CMV/ pcDNA5	GBP: GFP binding protein[5]
pMDLg/pRRE		CMV / pMD	3rd generation lentiviral packaging plasmid; Contains Gag and Pol; also requires pRSV-Rev (Addgene#12253) and envelope expressing plasmid (Addgene#12259)
pRSV-Rev		pREP / pRSV-Rev	3rd generation lentiviral packaging plasmid; Contains Rev; also requires pMDLg/pRRE (Addgene#12251) and envelope expressing plasmid (Addgene#12259)
HSPD1/HSPE1 promoter sequence short ver	*KpnI*-HSPD1 promoter sequence short ver-*SacI*-Luc(+)-SV40 late poly (A) signal-Enhencer	HSPDE1/pGL3 enhencer	TGCGCTCGGCGAGACAGGTCGTCGGCGGCGAGTGAGGGACAGAGTGCAGGGCGCACACCGCAATGAGCCCGTGTCCCCTCCCTCCGCCTCTACTCCCGCCCCGCGGCACCGCGTGTGCAGGCAGCTCCCACCCACTTCCCGTCAGCCCGGGCCCTGCAATCTGCACACCCTGCGCGCGAGCCCCGCCCCTCCCTACCCGCGCAGGGTGTGCTAGCGCGCTCAGCCCTCTCCGGCCGGCTTAGTCTAGTTCCCGGGCCTCGCTCGGTTCCAGAACTTTCCAGAAAATGCCGCGCTCCCTACGGCTCAAGGGTCAAATCGCGTCATTTCCGGGAGGGGACGAAGGGGTAGTTCTTTCACCTCGGCTGGGCGCCTAGAAAAGCCTAGAAACAGCTCCTTTTTTCTTCCGCCTCCGAGTCTTCGCGTCAGCGTCCTGCGCAGGGCCCTTGGGGCGAATCGCGGTGCGCGTCGGGGCGACCGCCCTCCCTCCCTGGGAGGGGCGAGGGGGCTAGCGGCGACCGCTGGGGCGAGCGCGCCTGCGCGCTGGGTGATTTTTTCACGTGTCGCCAGGGCCGGACTGCGAG (HSPD/E1 promoter sequence short ver)
NTS-pHluorin2	*KpnI*-NTS-*BamHI*-pHluorin2-Stop-*NotI*	CMV/ pcDNA5	NTS:MPEPAKSAPAPKKGSKKAVTKAQKKDGKKRKRSRKESYSVYVYKVLKQVHPDTGISSKAMGIMNSFVNDIFERIAGEASRLAHYNKRSTITSREIQTAVRLLLPGELAKHAVSEGTKAVTKYTSAK (nuclear targeting sequence) and pHluorin2 is from addgene (Plasmid #73794)
MTS-Myc-mCherry	*KpnI-MTS-BamHI-myc-dsRed-Stop-NotI*	CMV/ pcDNA5	Mitochondrial targeting sequence (MTS): MLATRVFSLVGKRAISTSVCVRAH[4]<br><br>Myc epitope tag: EQKLISEEDL

The nuclear export sequence, NES (LQLPPLERLTLD), was derived from residues 6–17 of the HIV-1 Rev protein. Protein processed size during translocation was obtained by programs: ApE (https://jorgensen.biology.utah.edu/wayned/ape/)

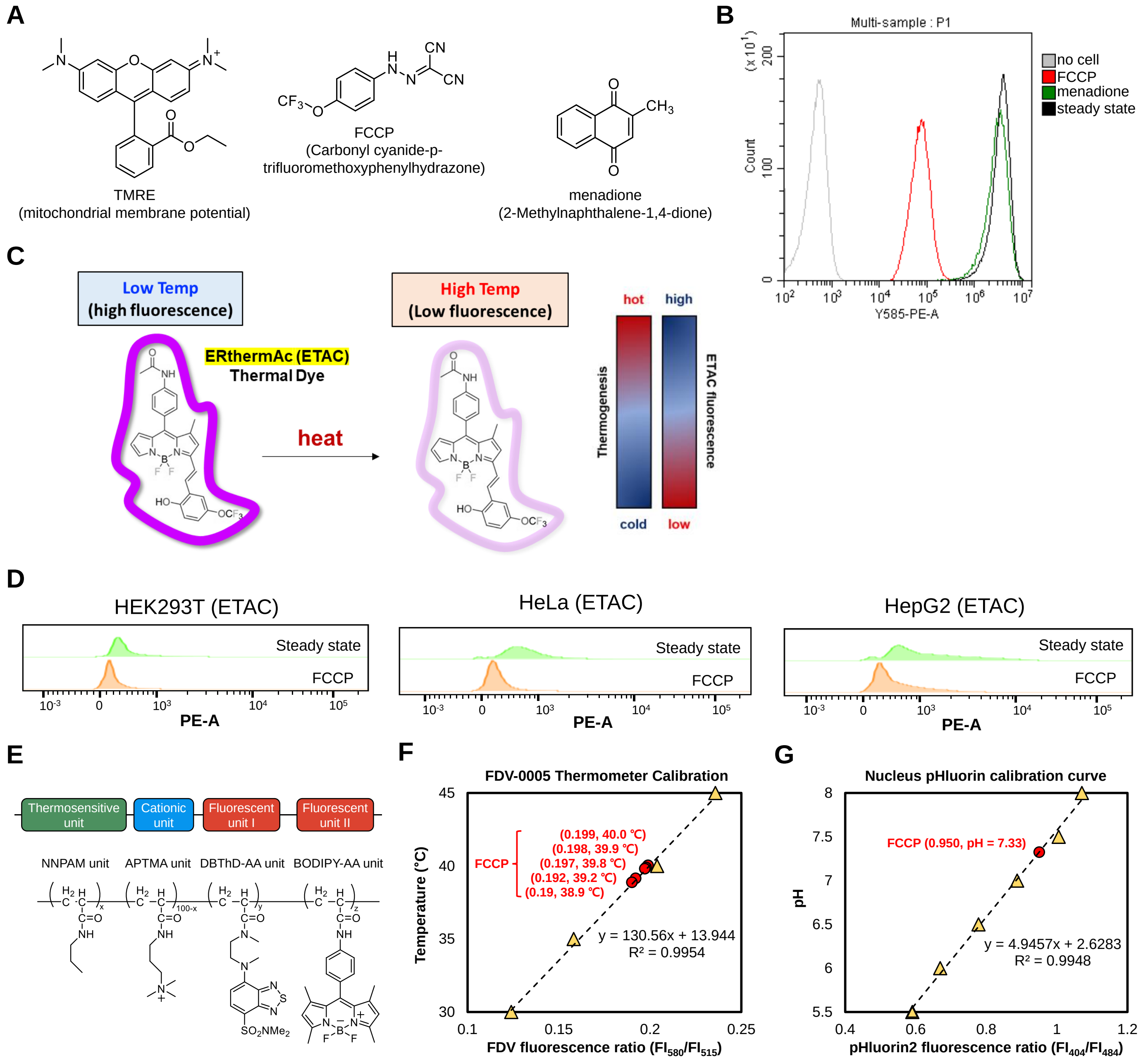

Figure S1. Confirm the intracellular thermogenesis by two different thermometers. (**A**) Chemical structures of TMRE, FCCP, and menadione. (**B**) Flow cytometry analyses and its histogram of HEK293T cell suspensions treated with either TMRE, FCCP, or menadione. (**C**) Schematic illustration of temperature measurement by ETAC. (**D**) ETAC fluorescence intensity after FCCP (100 µM, 1 h) treatment. Decreased ETAC fluorescence intensity under the indicates increased local temperature at the ER membrane. Flow cytometry analysis results of ETAC fluorescent signal under the two conditions (steady-state or 100 µM FCCP, 1 h), in HEK293T, HeLa and HepG2 cell lines. Cell count is shown on the y-axis, while fluorescent signal intensity (PE-A) is shown on the x-axis. (**E**) The polymer structure of the organic thermometer "FDV-0005". (**F**) Intracellular temperature calibration was conducted using the FDV-0005 organic thermometer. The FDV-0005 was used at a dilution of 0.05% w/v in 5% glucose solution. Measurements for calibration curve were performed within a temperature range of 30 °C to 45 °C (n = 5). To determine the temperature elevation induced by FCCP, cells were treated with 100 µM FCCP after FDV-0005 incubation for 10 minutes (n = 5). Red dots represent the FCCP-treaded samples. (**G**) Measurement of nuclear pH under the FCCP condition. The fluorescent protein-based pH sensor pHluorin2 was used. The HEK293T cells were incubated at 37 °C and 5% CO_2 in a humidified incubator and 100 µM FCCP was used for this assay.

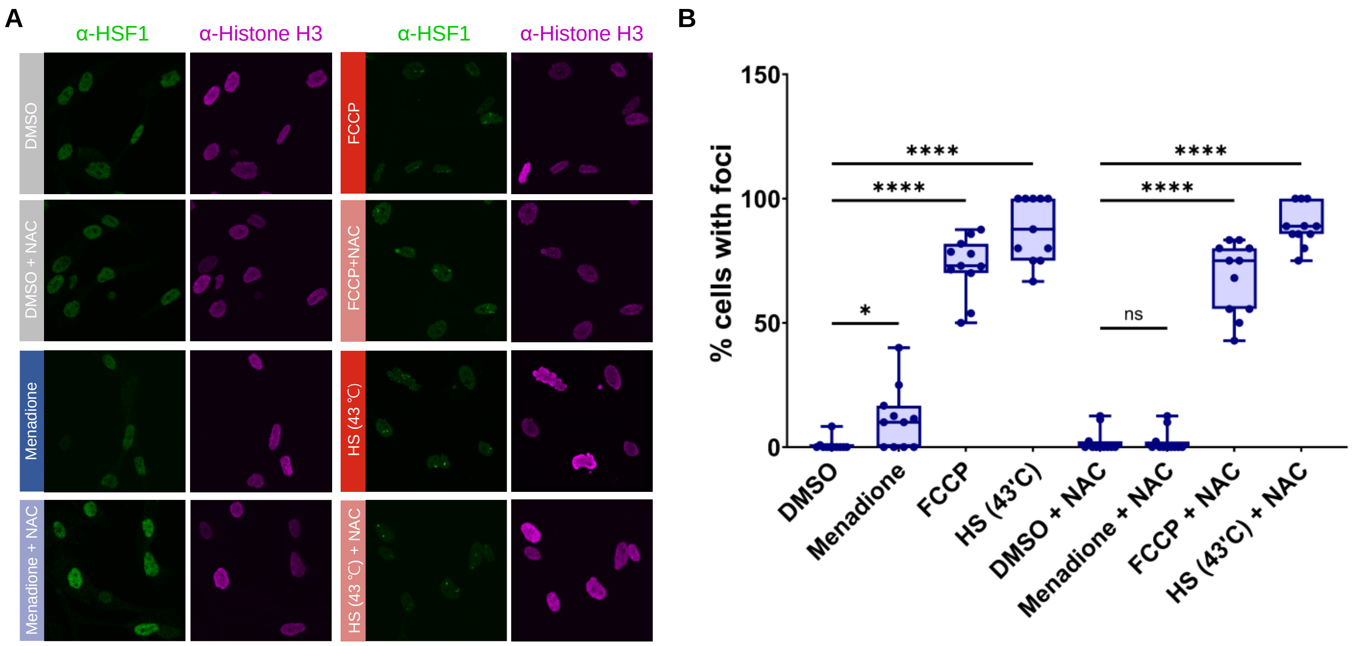

Figure S2. Endogenous HSF1 foci detection in the MCF10A cells with various stress conditions. (**A**) Z-stack images of endogenous HSF1 (anti-HSF1) and Histone H3 (anti-Histone H3) were obtained by 3D confocal imaging with activated MCF10A cells treated with either DMSO or FCCP (100 µM, 1 h) or menadione (30 µM, 30 min), or subjected to heat shock (43 °C, 1 h), and with or without co-treatment with NAC (5 mM, 1 h). (**B**) Percentage of cells with foci was quantified by evaluating 50-100 cells. Boxes indicate the quartiles, whiskers range from minimal to maximal values, and dots represent individual data points plotted on the box. Statistical analysis was conducted using an unpaired two-tailed t-test and the significance level was denoted as (*$p < 0.05$, ****$p < 0.0001$, ns, not significant).

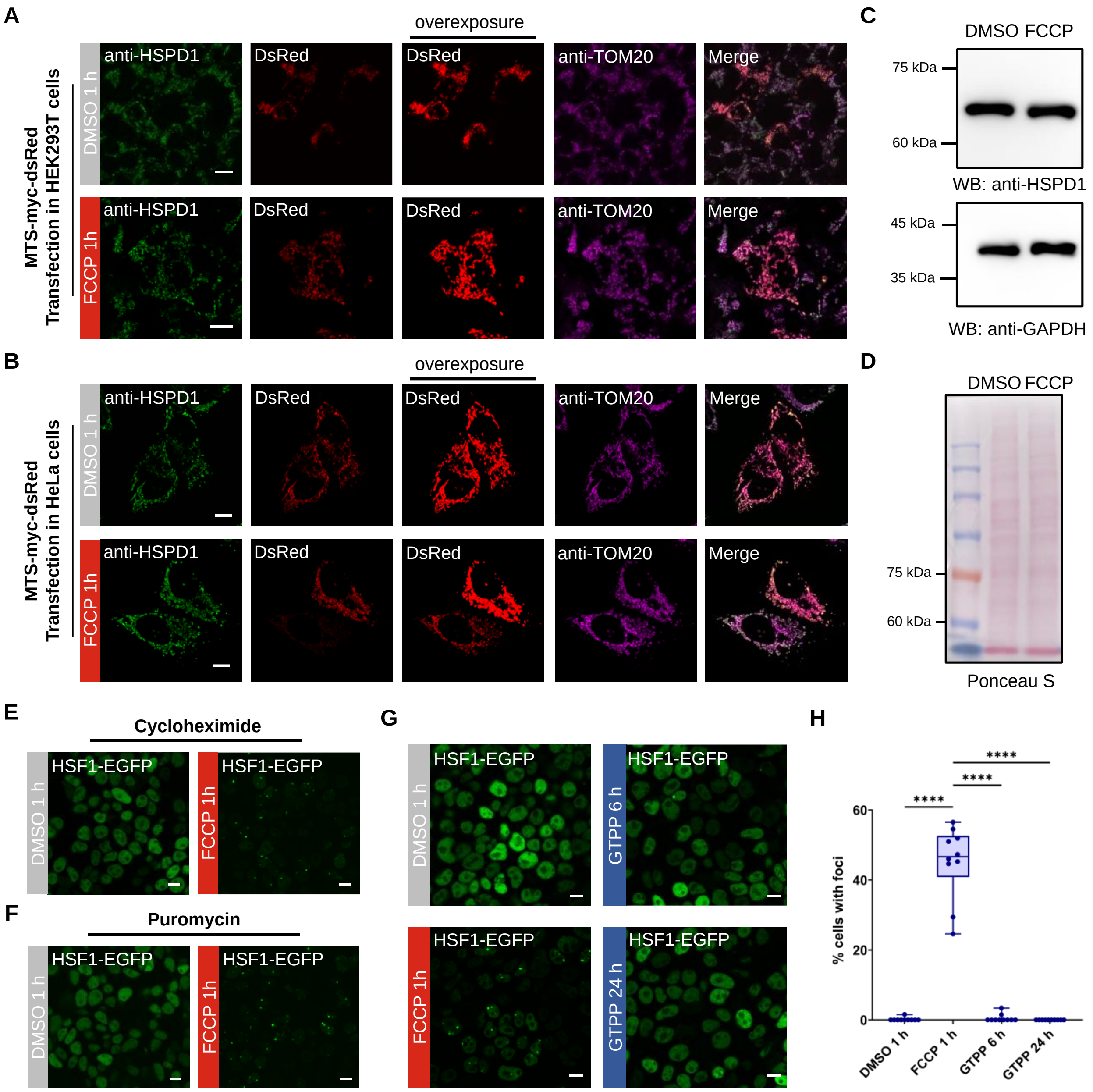

Figure S3. The formation of HSF1 foci induced by FCCP is independent of accumulated mitochondrial proteins. Subcellular localization of transiently expressed MTS-myc-dsRed and endogenous HSPD1 were visualized in HEK293T (**A**) and HeLa cells (**B**) by immunofluorescence with anti-Myc and anti-HSPD1 antibodies. Scale bar = 10 µm. (**C**) Western blot analysis of endogenous HSPD1 under the DMSO (control) or FCCP (100uM, 1hr)-treated sample using an anti-HSPD1 antibody. (**D**) Ponceau staining of the same membrane in (**C**) was used as reference. (**E-F**) Foci formation of HSF1-EGFP in FCCP-treated cells under protein synthesis blockade by cycloheximide (**E**) and puromycin (**F**) was observed. Cycloheximide (35 µM) and puromycin (35 µM) were pre-treated for 2 h followed by addition of DMSO or FCCP (100 µM) for 1 h. (**G**) Confocal images of HSF1-EGFP foci formation were obtained after treatment with either DMSO or FCCP (100 µM) for 1 h or GTPP (10 µM) for 6 h or 24 h. Scale bar = 10 µm. (**H**) Quantification of percentage of the HSF1 foci formation cells was performed by evaluating 500-640 cells per condition. Boxes indicate the quartiles, whiskers range from minimal to maximal values, and dots represent individual data points plotted on the box. Statistical analysis was conducted using a one-way analysis of variance (ANOVA) with Tukey's test, and the significance level was denoted as (****$p < 0.0001$).

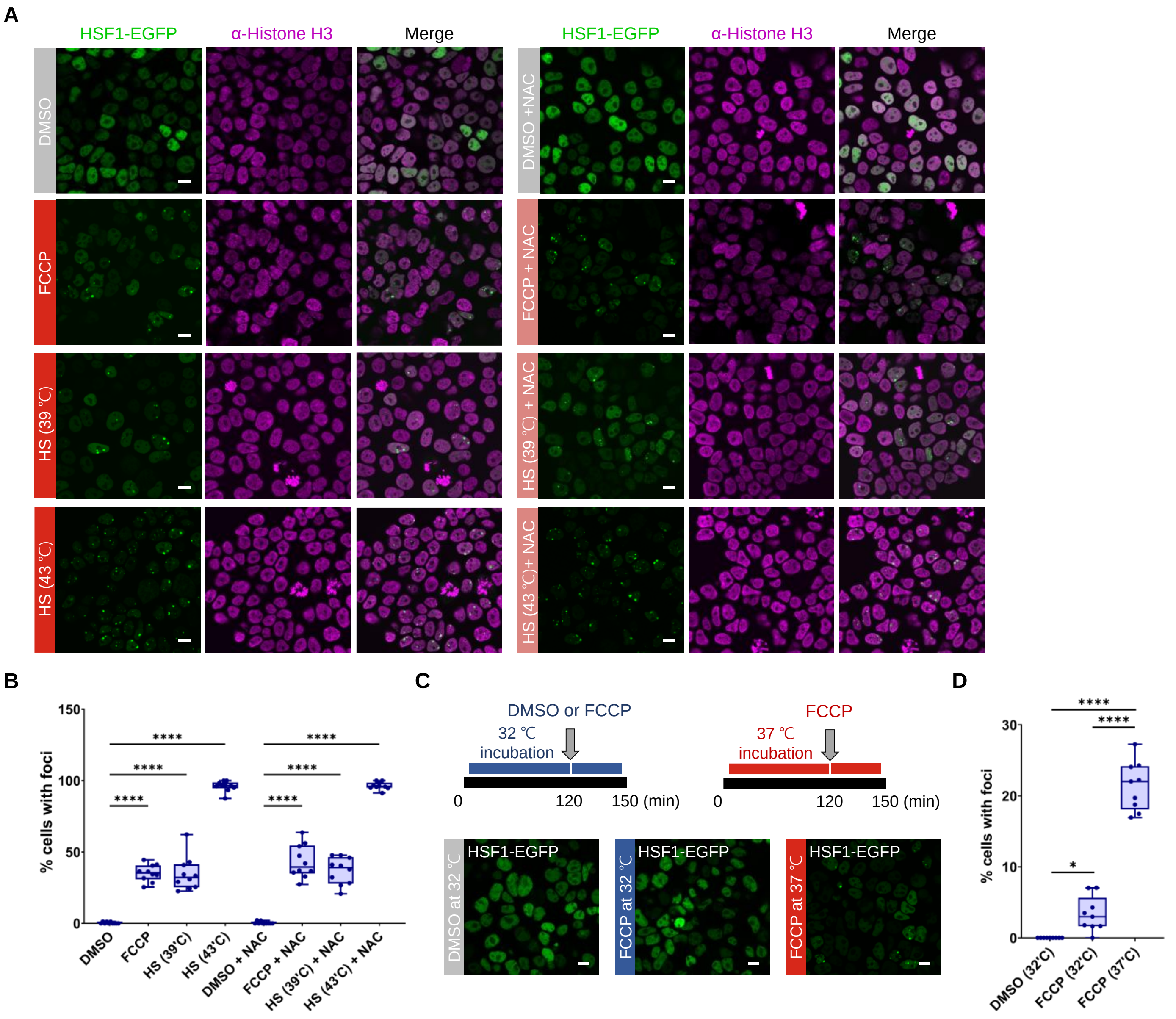

Figure S4. Quantification of the HSF1-EGFP foci in various stress conditions. (**A**) The level of HSF1-EGFP foci formation in the HSF1-EGFP stable cell line under the different conditions including DMSO, FCCP (100 μM, 1hr), 39 °C (1 hr), 43 °C (1 hr) were visualized by confocal imaging with immunofluorescence. Scale bar = 10 μm (**B**) Percentage of the cells with foci in the imaging results of (**A**) were analyzed, 450-750 cells per condition were evaluated for quantification. (**C**) The formation of HSF1-EGFP foci induced by FCCP (100 μM, 1hr) at 32 °C was visualized by confocal imaging. Cells were pre-cooled at 32 °C in a humidified 5% CO_2 incubator for 2 h. FCCP-treated cells at 37 °C were used as positive control. Scale bar = 10 μm (**D**) Percentage of the cells with foci in the imaging results of (**C**) were analyzed. 450-750 cells per condition were evaluated for quantification. Boxes in (**B**) and (**D**) indicate the quartiles, whiskers range from minimal to maximal values, and dots represent individual data points plotted on the box. Statistical analysis was conducted using a one-way analysis of variance (ANOVA) with Tukey's test, and the significance level was denoted as (*$p < 0.05$, ****$p < 0.0001$).

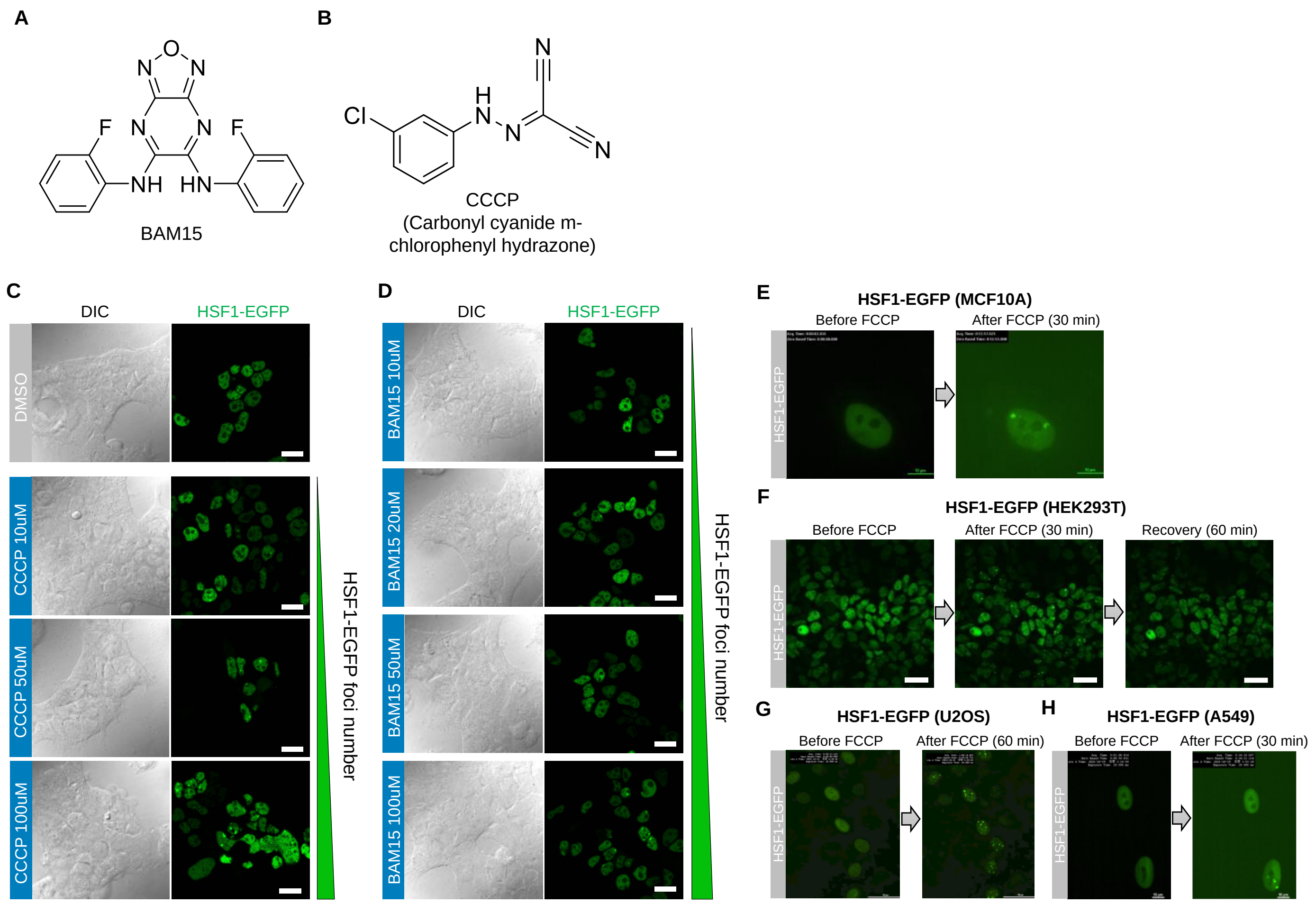

Figure S5. HSF1-foci formation in various protonophores and HSF1-EGFP stably expressed cell lines. (**A-B**) Chemical structures of BAM15 and CCCP. (**C**) Confocal images of HSF1-EGFP foci formation in HEK293T HSF1-EGFP-expressing cells following treatment with various CCCP concentrations (10, 50, and 100 µM; 1 h incubation). Scale bar = 20 µm. (**D**) Confocal images of HSF1-EGFP foci formation in HEK293T HSF1-EGFP-expressing cells under treatment with various BAM15 concentrations (10, 20, 50, and 100 µM; 1 h incubation). Scale bar = 20 µm. (**E**) Live-cell imaging of HSF1-EGFP foci formation following FCCP treatment (100 µM, 30 min) of HSF1-EGFP-expressing MCF10A cells. A real-time video recording is shown in **supplemental Movie 3,4**. Scale bar = 10 µm. (**F**) Live-cell imaging of HSF1-EGFP foci formation following FCCP treatment (100 µM, 30 min) of HSF1-EGFP-expressing HEK293T cells. A real-time video recording is shown in **supplemental Movie 5-7**. Scale bar = 50 µm. (**G**) Live-cell imaging of HSF1-EGFP foci formation following FCCP treatment (100 µM, 5 min) of HSF1-EGFP-expressing U2OS cells. A real-time video recording is shown in **supplemental Movie 8,9**. (**H**) Live-cell imaging of HSF1-EGFP foci formation following FCCP treatment (100 µM, 30 min) of HSF1-EGFP-expressing A549 cells. All live cell experiments were conducted at 37 °C in a humidified 5% CO_2 incubator. A real-time video recording is shown in **supplemental Movie 10**. Scale bar = 10 µm.

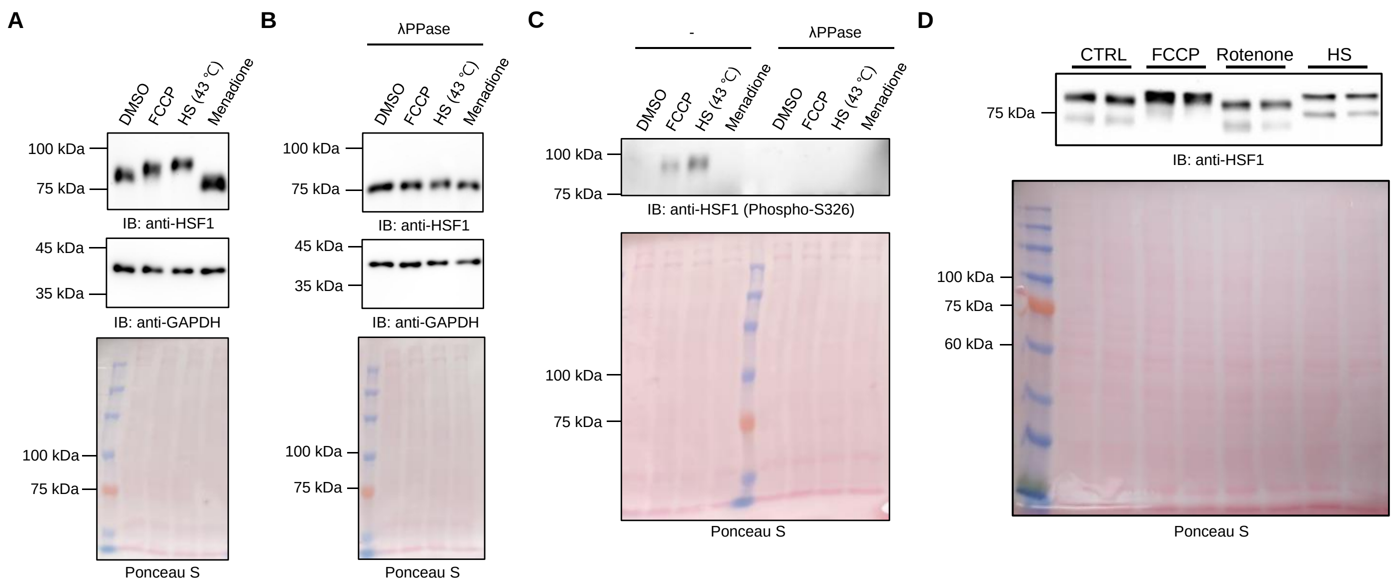

Figure S6. FCCP treatment induces phosphorylation of endogenous HSF1 (**A-B**) Western blot analysis of endogenous HSF1 following treatment with FCCP (100 µM, 1 h) or menadione (30 µM, 30 min) or heat shock (43 °C, 1 h) in MCF10A cells. DMSO-treated cells were used as the control. Lysates were incubated with only buffer condition (**A**) or with lambda protein phosphatase (λPPase) solution (**B**) for 30 minutes before gel running. GAPDH and Ponceau S staining were used as references. (**C**) Detection of phosphorylated HSF1 (phospho-S326) in the same stress conditions used in **A**. Ponceau S staining was used as a reference. (**D**) Western blot analysis of recombinant HSF1-EGFP modification, following treatment with FCCP (100 µM, 1 h) or heat shock (43 °C, 1 h) in MCF10A cells. Ponceau S staining was used as reference.

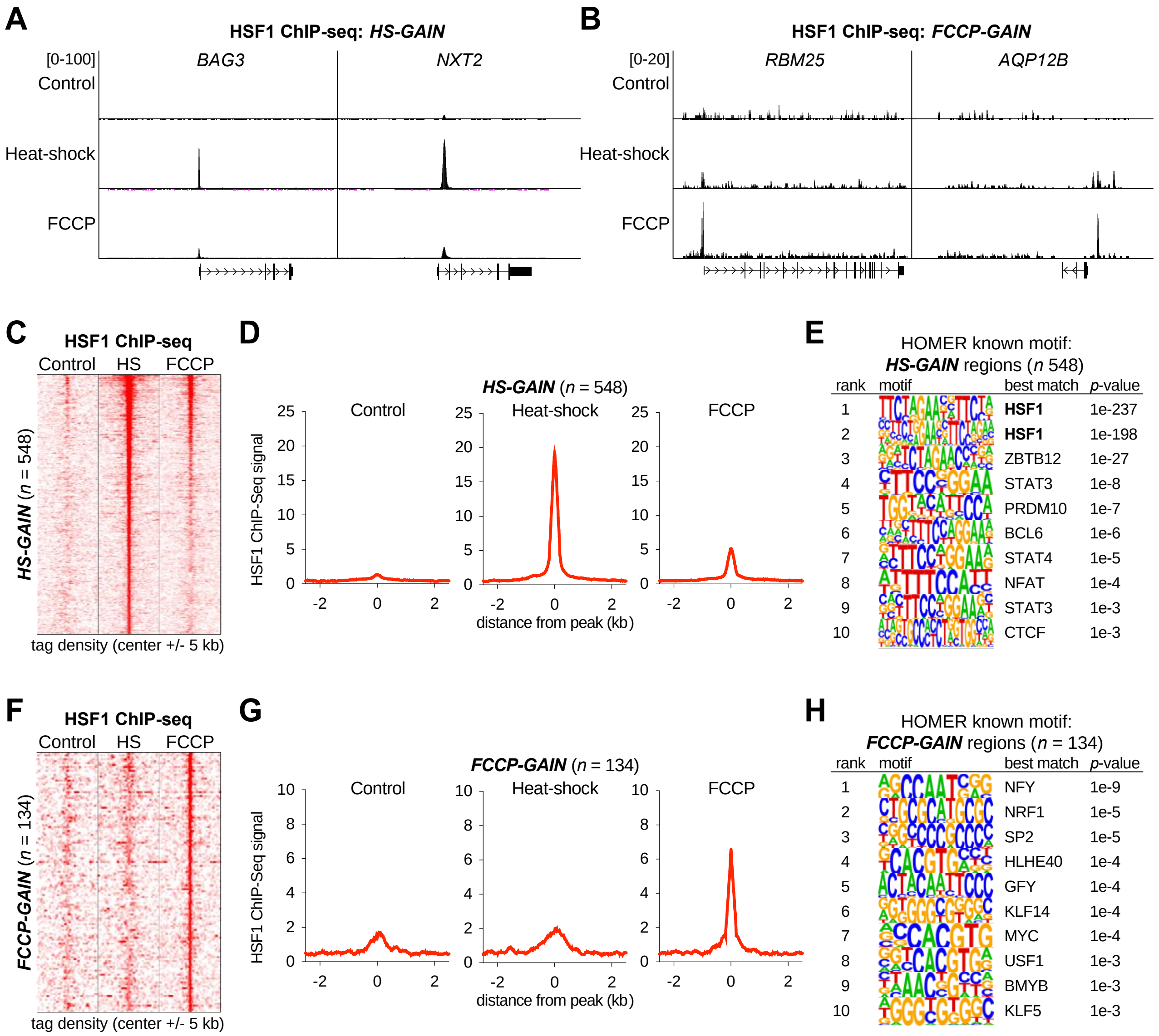

Figure S7. Chip-seq and RNA-seq analysis of HSF1 under heat shock-specific and FCCP treatment-specific *GAIN*. (A–B) Representative HSF1 ChIP-seq profiles at the (A) *BAG3* and *NXT2*, and (B) *RBM25* and *AQP12B* loci. (C) Density plots of HSF1 ChIP-seq, signal enrichment at a 5-kb region around the center of heat shock-specific bound regions. Each row represents a single region ($n = 548$). (D) Analysis of the ChIP-seq signals (shown in C) overactive promoters and enhancers. The scale of all graphs was calculated using the distance of the peaks (kb). (E) Motif analysis (HOMER) based on HSF1 ChIP-seq results showing enriched motifs in *HS-GAIN* peaks. Only the top 10 statistically significant motifs are shown in the logo plots. (F) Density plots of HSF1 ChIP-seq and signal enrichment at a 5-kb region around the center of the FCCP treatment-specific bound regions. (G) Analysis of the ChIP-seq signals (shown in **F**) overactive promoters and enhancers. The scales of all graphs were calculated using the distance of the peaks (kb). (H) Motif analysis (HOMER) based on HSF1 ChIP-seq results showing enriched motifs in *FCCP-GAIN* peaks. Only the top 10 statistically significant motifs are shown in the logo plots.

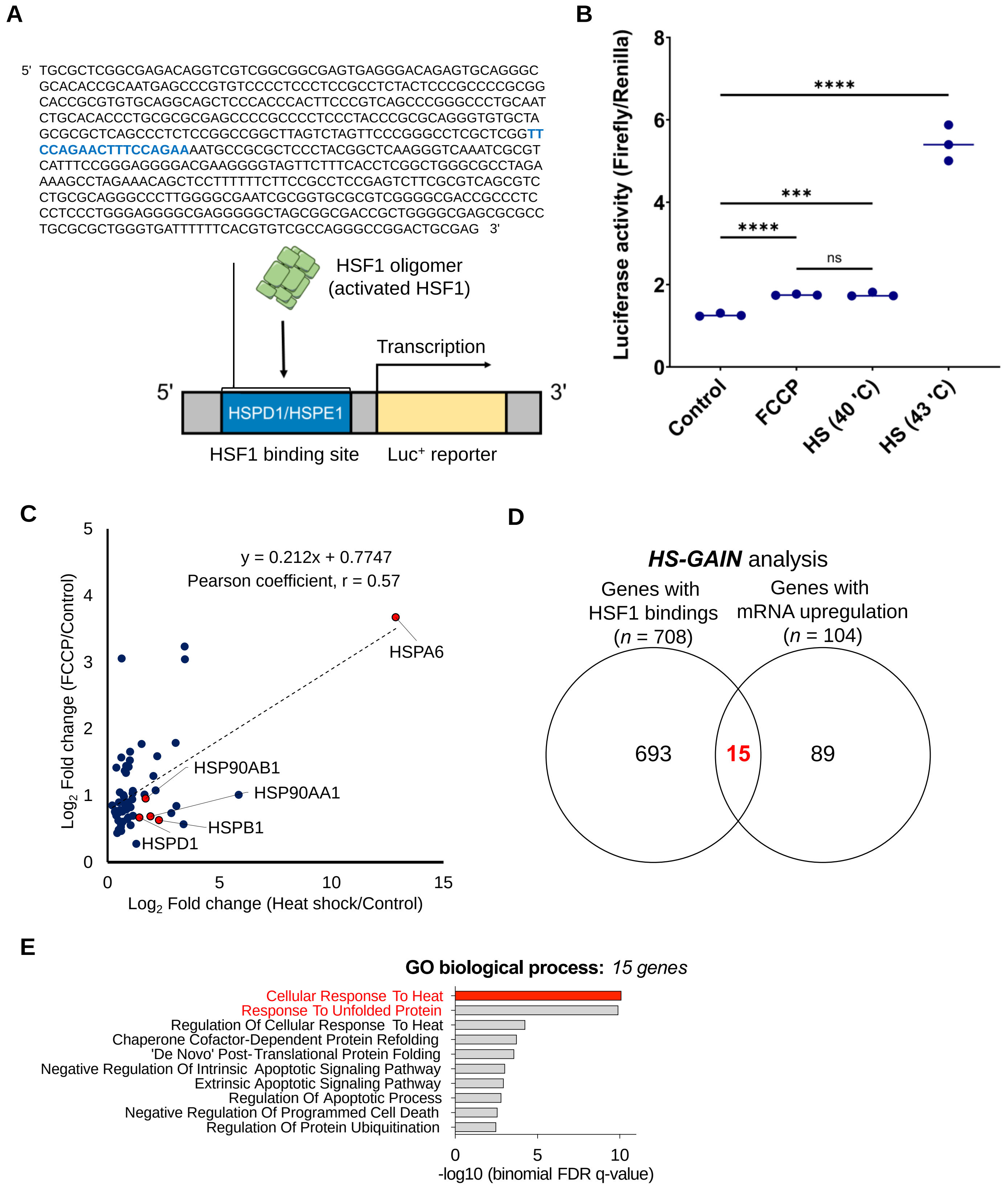

Figure S8. The results of luciferase assay with HSF1 binding sequence in *HSPD1/HSPE1* & transcription expression correlation of common-GAIN genes under Heat shock and FCCP treatment (**A**) The HSF1 binding sequence of HSPD1/HSPE1 and schematic representation of the HSPD1-luciferase assay are illustrated. The HSF1 binding site was marked with light blue in the sequence. (**B**) HSPD1/HSPE1 promoter luciferase assay results in HEK293T cells after FCCP treatment (100 μM, 1 h), heat shock at 40 °C

Figure S8. (continued) or 43 °C for 1 h. All experiments were conducted using live cells incubated at 37 °C, in a humidified 5% CO_2 incubator. The middle line of the individual value plot indicates the median value, and each dot indicates individual data points. Statistical analysis was conducted using an unpaired two-tailed t-test and the significance level was denoted as (***p < 0.001, ****p < 0.0001, ns, not significant). (**C**) Scatter plot of mRNA expression level of Common-GAIN genes (61 gene) under the heat shock (x-axis) and FCCP treatment condition (y-axis). In each condition, Log2 fold change values of mRNA expression over the control sample were presented in the graph. Original data for this graph is shown in the **Supporting Information**. (**D**) Venn diagram showing the overlap (*n* = 15) of the HSF1-bound genes (*n* = 708) and mRNA upregulated genes (*n* = 104) under heat shock-specific conditions. (**E**) Gene Ontology (GO) analysis of the biological processes associated with the identified 15 upregulated HSF1-bound genes (as shown in **D**).

Supplemental Movie legends

Movie 1. Live imaging of U2OS cells stably expressing HSF1-EGFP shown in the Fig. 3B, 0 – 10 min: normal growth media, 10 – 40 min: FCCP-treated media, 40 – 120 min: normal growth media.

Movie 2. Wide view of live cell imaging in Fig. 3B.

Movie 3. Live imaging of MCF10A cells stably expressing HSF1-EGFP shown in the Fig. S5E, 0 – 30 min: normal growth media, 30 – 82 min: FCCP-treated media.

Movie 4. Wide view of live cell imaging in Fig. S5E.

Movie 5. Live imaging of HEK293T cells stably expressing HSF1-EGFP in normal growth media for 30 min shown in Fig. S5F.

Movie 6. (Continued from Movie 5) Live imaging of HEK293T cells stably expressing HSF1-EGFP in FCCP-treated media for 30 min shown in Fig. S5F.

Movie 7. (Continued from Movie 6) Live imaging of HEK293T cells stably expressing HSF1-EGFP in normal growth media for 30 min shown in Fig. S5F.

Movie 8. Live imaging of U2OS cells stably expressing HSF1-EGFP shown in Fig. S5G, 0 – 30 min: normal growth media, 30 – 67 min: FCCP-treated media.

Movie 9. Wide view of live cell imaging in Fig. S5G.

Movie 10. Live imaging of A549 cells stably expressing HSF1-EGFP shown in Fig. S5H, 0 – 30 min: normal growth media, 30 – 35 min: FCCP-treated media, 35 – 67 min: normal growth media.

Movie 11. Live imaging of HEK293T cells stably expressing HSF1-EGFP after FCCP treatment under hypoxia condition (1% O_2) shown in Fig. 4B.

References

(1) Kriszt, R.; Arai, S.; Itoh, H.; Lee, M. H.; Goralczyk, A. G.; Ang, X. M.; Cypess, A. M.; White, A. P.; Shamsi, F.; Xue, R. Optical visualisation of thermogenesis in stimulated single-cell brown adipocytes. *Scientific Reports* **2017**, *7* (1), 1383.

(2) Uchiyama, S.; Tsuji, T.; Ikado, K.; Yoshida, A.; Kawamoto, K.; Hayashi, T.; Inada, N. A cationic fluorescent polymeric thermometer for the ratiometric sensing of intracellular temperature. *Analyst* **2015**, *140* (13), 4498-4506.

(3) Kim, H. R.; Yim, J.; Yoo, H. B.; Lee, S. E.; Oh, S.; Jung, S.; Hwang, C. I.; Shin, D. M.; Kim, T.; Yoo, K. H.; et al. EVI1 activates tumor-promoting transcriptional enhancers in pancreatic cancer. *NAR Cancer* **2021**, *3* (2), zcab023. DOI: 10.1093/narcan/zcab023.

(4) Fornuskova, D.; Stiburek, L.; Wenchich, L.; Vinsova, K.; Hansikova, H.; Zeman, J. Novel insights into the assembly and function of human nuclear-encoded cytochrome c oxidase subunits 4, 5a, 6a, 7a and 7b. *Biochemical Journal* **2010**, *428* (3), 363-374. DOI: 10.1042/bj20091714 (acccessed 5/5/2024).

(5) Rothbauer, U.; Zolghadr, K.; Tillib, S.; Nowak, D.; Schermelleh, L.; Gahl, A.; Backmann, N.; Conrath, K.; Muyldermans, S.; Cardoso, M. C.; et al. Targeting and tracing antigens in live cells with fluorescent nanobodies. *Nat Methods* **2006**, *3* (11), 887-889. DOI: 10.1038/nmeth953.
