# Supplementary material for: Mitochondrial Thermogenesis Can Trigger Heat Shock Response in the Nucleus
Source: ACS Cent Sci. 2024 Jun 3;10(6):1231–41. doi: 10.1021/acscentsci.3c01589 (PMC11212142; doi:10.1021/acscentsci.3c01589)
Supplement: Supplementary file 3 — oc3c01589_si_003.pdf [file oc3c01589_si_003.pdf]

oc-2023-015892.R1

Name: Peer Review Information for "Mitochondrial thermogenesis regulates heat-shock response in the nucleus"

First Round of Reviewer Comments

Reviewer: 1

Comments to the Author

The authors report an interesting study where they show that the heat generated from mitochondria, under FCCP-stimulated conditions, can lead to the activation of the heat shock response (HSR) pathway, so that HSF1 forms foci in the cell nuclei.

Overall, the results are interesting and potentially impactful: on the technical side, the HSF1 clustering assay they developed provides a creative way to detect cell heat. On the scientific side, their results show that mitochondria, at least under the FCCP-stimulated conditions, can generate enough heat to modulate cellular functions, thus an interesting pathway that may be considered in future studies. I thus recommend publication after the authors consider the comments below.

1. The behavior of endogenous HSF1 is important, and the data should be better quantified. For example, for data like Fig 1c, it would be helpful to do statistics, e.g., the number of foci per cell for the different conditions.

2. The time of treatment with the different conditions should be mentioned in the text. Now they are hidden in the caption.

3. Since their temperature measurements suggest FCCP stimulation raises the temperature to 39 C, it seems they should also compare results with heat shocks at 39 C. Right now all heat shock experiments are at 43 C.

4. The authors tested many conditions to show that FCCP-induced HSF1 foci were due to heat release. However, there is another more direct test that needs to be examined: Maintain the cells at a relatively low temperature, like 32 C, and then add FCCP. In this case, FCCP should still induce

most of the other effects in the cell, but now it seems will not be able to increase the cell temperature to >37 C, so this sounds like could be a better control.

5. The authors discuss that in FCCP-treated cells, HSF1 western blot showed slower migrations, and they attributed it to phosphorylation. This discussion is not convincing, e.g., in Fig S3B, no significant size differences are found vs the control. Also, phosphorylation tends to generate smeared bands or multiple bands, unless it is well-defined for a particular state?

6. The discussion of "which leads to which" may be confusing in their discussion of the RNA-seq data. HSF1 is a transcription factor, so its activation could have led to the activation of other genes?

7. For the gene expression fold-change data, it may be useful to plot the fold-change in FCCP-treated cells versus heat-shock cells for each gene, then a positive correlation may be identified.

Reviewer: 2

Comments to the Author

In human cells, mitochondrial dysfunction induces a stress-induced change in gene expression. The heat stress response is part of this reaction. Previous studies have elucidated mechanisms by which mitochondrial proteins (precursor proteins) can trigger reactions in the nucleus. The best-characterized response program is that of C elegans where the transcription factor ATFS-1 is equipped with a mitochondrial signal sequence. Mitochondrial dysfunction prevents the mitochondrial translocation of this factor which therefore ends up in the nucleus to trigger a mitochondrial stress response (Nargund et al. 2012. Science 337, 587-590). Similar response pathways were described in human cells.

In this study, the authors use the well-characterized ionophore FCCP. By uncoupling the proton gradient of the mitochondrial inner membrane, FCCP leads to mitochondrial dysfunction and the increased production of reactive oxygen species (ROS). Uncoupling leads to heat production in cells (shown also in Figure S1) and triggers the heat shock response (Figure 1+3). The authors present some evidence that ROS production is not essential for induction of the heat shock response, which is in line with previous studies. Based on their results, they propose that the production of heat in mitochondria triggers the heat shock response. This is an interesting hypothesis. However, no direct evidence is provided here. Previous studied delineated the stress response pathways in detail. For example, a recent publication by Munch (Sutandy et al. 2023 Nature 618, 849-854) showed that the binding of non-imported mitochondrial proteins to the

HSP40 protein DNAJA1 is crucial for the response to mitochondrial dysfunction (including the HSR). The relevance of mitochondrial precursor proteins was also reported before in many other studies. FCCP induces the accumulation of mitochondrial precursor proteins in the cytosol. Rhee and coworkers now suggest that thermogenesis, but not accumulating precursor proteins, trigger the heat shock response. However, as it stands, evidence for this claim is missing.

Specific problems:

1. The authors need to study conditions at which the effect on heat production can be uncoupled from that on protein import. According to their hypothesis, inhibition of respiration should suppress the effect of FCCP as this would prevent thermogenesis.

2. Along the same lines: can the authors repress the synthesis of mitochondrial precursor proteins so that their accumulation does not trigger the HSR upon FCCP treatment?

3. In general, FCCP is a very harsh treatment and many studies in the context of PINK-Parkin showed that the FCCP-induced effects are more extreme than those detected under physiological conditions. Thus, under the normal conditions of mitochondrial function or dysfunction, does mitochondrial thermogenesis for the HSR really matter? It seems unlikely to this referee that outside of brown adipose tissue cells, heat production from mitochondria will be of any physiological relevance.

Author's Response to Peer Review Comments:

We thank the reviewers for providing extremely helpful and constructive for improving our manuscript. We have attached our point-by-point responses to each reviewer's comments.

Reviewers' Comments

Reviewer #1 (Remarks to Author)

The authors report an interesting study where they show that the heat generated from mitochondria, under FCCP-stimulated conditions, can lead to the activation of the heat shock response (HSR) pathway, so that HSF1 forms foci in the cell nuclei.

Overall, the results are interesting and potentially impactful: on the technical side, the HSF1 clustering assay they developed provides a creative way to detect cell heat. On the scientific side, their results show that mitochondria, at least under the FCCP-stimulated conditions, can generate enough heat to modulate cellular functions, thus an interesting pathway that may be considered in future studies. I thus recommend publication after the authors consider the comments below.

We thank the reviewer for acknowledging the impact and comprehensiveness of our work. We have fully addressed each of the major suggestions made by the reviewer, as outlined below.

1 (Reviewer 1-1). The behavior of endogenous HSF1 is important, and the data should be better quantified. For example, for data like Fig 1c, it would be helpful to do statistics, e.g., the number of foci per cell for the different conditions.

Response: We appreciate the suggestion to quantitatively compare the formation of HSF1 foci under various stress conditions with a statistical manner. As suggested by the reviewer, we calculated the endogenous HSF1 foci number per cell (**Figure 1C-D**, below) and the percentage of cells with foci (**Figure S2**, below) using MCF10A cells. In this experiment, we conducted 3D confocal imaging with 50-100 cells (over the 10 different field of view images) in each condition and quantified the cells after obtaining Z-stack image collections. As shown in **Figure 1C** and **D**, we observed that the induced HSF1 foci numbers by FCCP (foci per cell: ~6, p value <0.0001) and by heat shock condition at 43 °C (foci per cell: ~12, p value <0.0001) was significant compared to DMSO-treated cells (foci per cell: 0). The cell population that has HSF1-GFP foci were significantly increased in FCCP-treated sample (~70%, p value <0.0001) and in heat shock (43 °C) treated sample (~88%, p value <0.0001) compared to the DMSO-treated sample (**Figure S2A** and **B**). In both experiments, the increased HSF1 foci numbers and cell population with HSF1 foci formation by FCCP and by heat shock were not affected by co-treatment with N-acetyl-L-cysteine (NAC), which is an antioxidant molecule. Notably, we found that the level of HSF1 foci formation induced by menadione (foci per cell: ~0.9, percentage of cells with foci: ~11%) was much lower than those values by FCCP and menadione-treated foci formation was largely affected by NAC co-treatment. All of these results are in good agreement with our previous imaging results of endogenous HSF1 foci formation with FCCP and menadione with or without NAC treatment (**Figure 1C**). P-value was obtained using unpaired two-tailed t-test. These results clearly support our claim that HSF1 activation induced by FCCP is independent of the ROS generation process. We thank the reviewer for giving us this opportunity to solidify our findings.

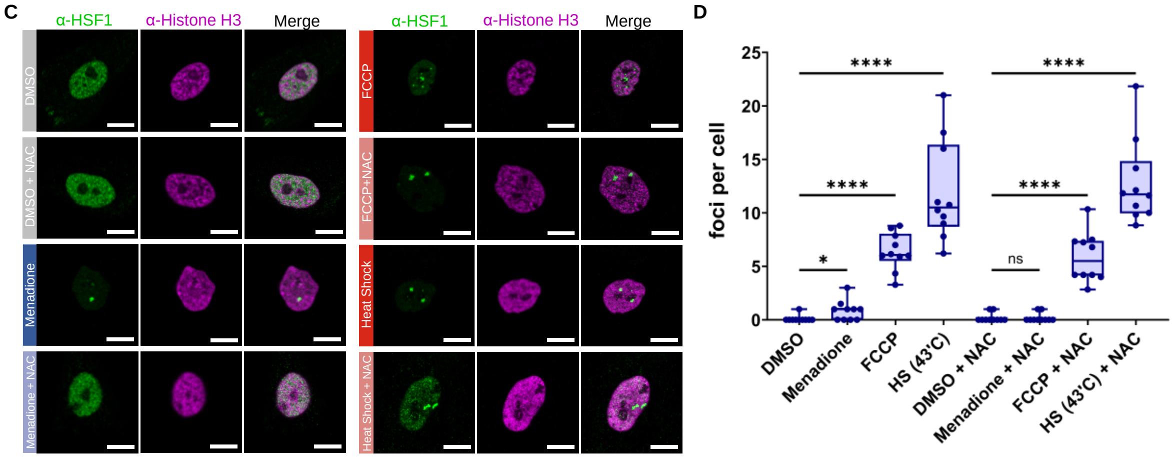

Figure 1 (updated). (**C**) Confocal images of endogenous HSF1 (anti-HSF1) and Histone H3 (anti-histone H3) activation in MCF10A cells incubated with either FCCP (100 µM, 1 h) or menadione (30 µM, 30 min), or subjected to heat shock (43 °C, 1 h), and with or without co-treatment with NAC (5 mM, 1 h). Scale bar = 10 µm. (**D**) The quantification of the number of foci per cell in both the control and various stress-induced cells corresponded to Figure 1C. In each condition, 50-100 cells were evaluated. Boxes indicate the quartiles, whiskers range from minimal to maximal values. Dots indicate individual data points plotted on the box. Statistical analysis was conducted using an unpaired two-tailed t-test and the significance level was denoted as (****p < 0.0001).

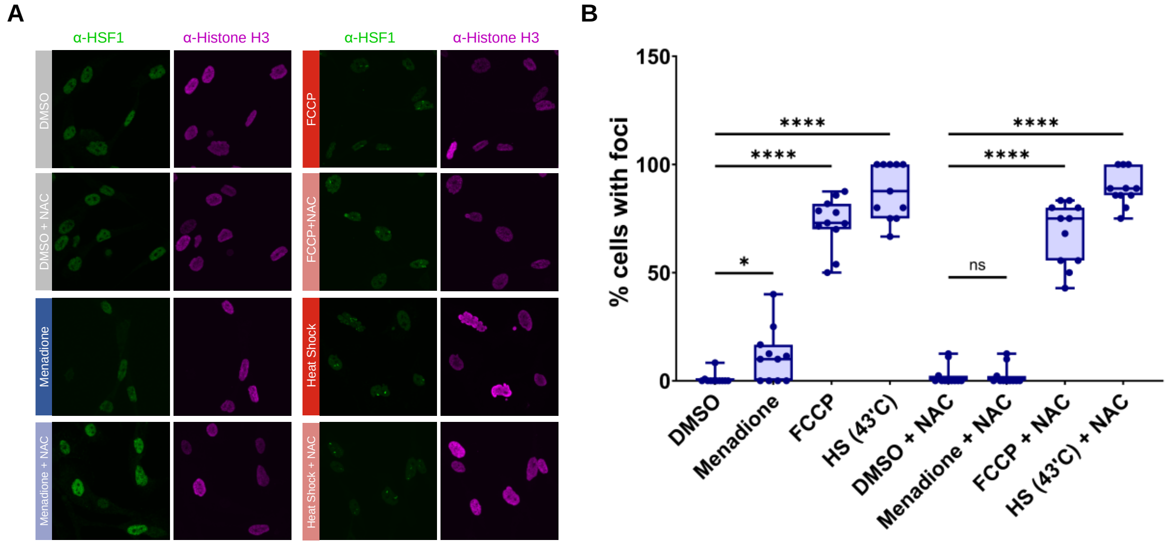

Figure S2. (**A**) Z-stack images of endogenous HSF1 in MCF10A were obtained in various stress conditions after 3D confocal imaging. Scale bar = 10 µm. (**B**) Percentage of cells with foci was analyzed, 50-100 cells were evaluated for quantification. Boxes indicate the quartiles, whiskers range from minimal to maximal values. Dots indicate individual data points plotted on the box. Statistical analysis was conducted using an unpaired two-tailed t-test and the significance level was denoted as (****p < 0.0001).

2 (Reviewer 1-2). The time of treatment with the different conditions should be mentioned in the text. Now they are hidden in the caption.

Response: We apologize for the missing information in the figure captions. We have updated the incubation time and concentration for reagents such as FCCP and menadione in **Figure 2,3a**, and **3c**.

3 (Reviewer 1-3). Since their temperature measurements suggest FCCP stimulation raises the temperature to 39 C, it seems they should also compare results with heat shocks at 39 C. Right now all heat shock experiments are at 43 C.

Response: Thank you for your insightful suggestion. As suggested by the reviewer, we conducted a mild heat shock experiment at 39 °C using the HSF1-GFP stable cell line (HEK293T). Consequently, we found that both the number of foci per cell and the percentage of the cells exhibiting foci in FCCP-treated cells increased to a similar level as HSF1 foci exposed at 39 °C (**Figure S4A, below**). We have also presented this data with statistical manner (unpaired two-tailed t-test) (**Figure S4B)** as suggested by reviewer in a previous comment.

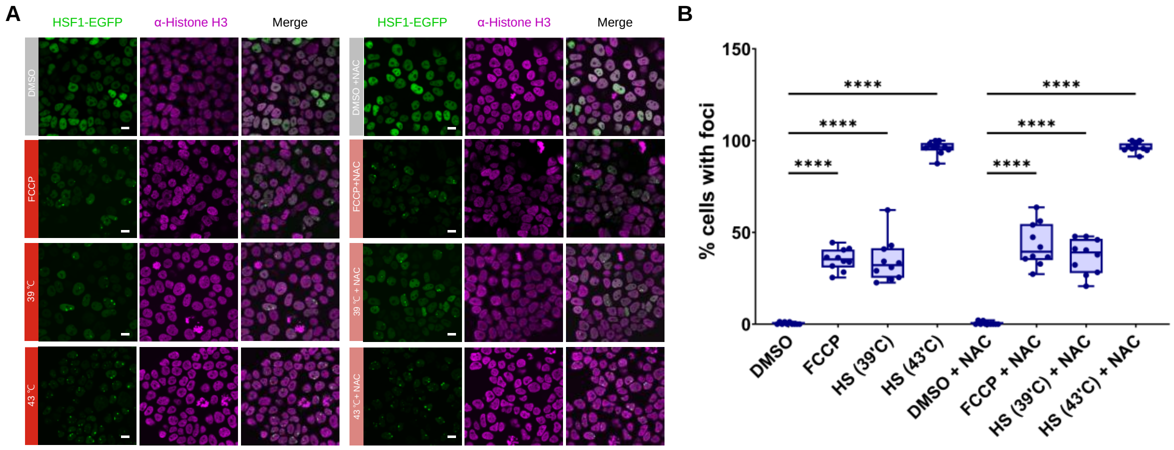

Figure S4 (updated). (**A**) The level of HSF1-EGFP foci formation in the stable cell line was visualized by confocal imaging with immunofluorescence. (**B**) Quantification of percentage of the cells with foci was performed by evaluating 450-750 cells. Boxes indicate the quartiles, whiskers range from minimal to maximal values, and dots represent individual data points plotted on the box. Statistical analysis was conducted using an unpaired two-tailed t-test, and the significance level was denoted as (****$p < 0.0001$).

During the revision, we also quantified our result of luciferase assay under FCCP treatment and heat shock (**Figure S8A**). In this result, we obtained the p-value using an unpaired two-tailed t-test. Consequently, we found that the luciferase activity of cells treated with FCCP showed no significant difference compared to cells subjected to heat shock (40 °C), whereas it was significantly higher compared to the control cells ($p < 0.0001$, **Figure S8B,** below). We also requantified the FDV results, which can indicate the intracellular temperature elevated by FCCP. Our requantified FDV result showed that FCCP raises the intracellular temperature from 38.9 °C to 40 °C (**Figure S1F**), which is in good agreement with those of our HSF1 foci measurement and HSPD1/HSPE1 promoter luciferase assay results (**Figure S8A** and **B**).

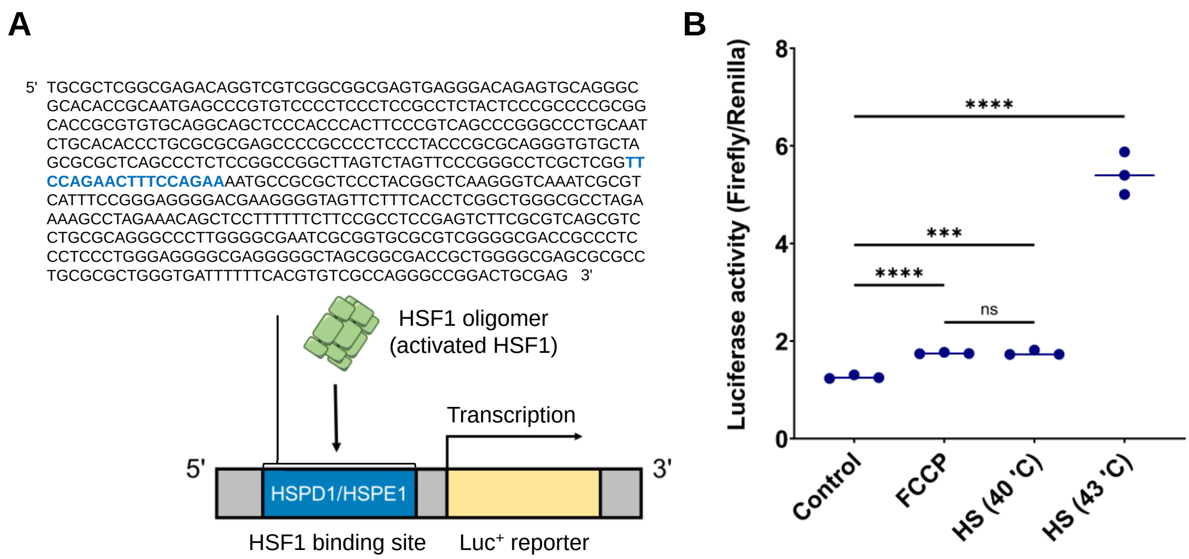

Figure S8 (updated). The results of luciferase assay with HSF1 binding sequence in *HSPD1/HSPE1* (**A**) The HSF1 binding sequence of HSPD1/HSPE1 and schematic representation of the HSPD1-luciferase assay are illustrated. The HSF1 binding site was marked with light blue in the sequence. (**B**) Luciferase assay results in HEK293T cells after FCCP treatment (100 μM, 1 h), heat shock at 40 °C or 43 °C for 1 h. All experiments were conducted using live cells incubated at 37 °C, in a humidified 5% CO_2 incubator. The middle line of the individual value plot indicates the median value, and each dot indicates individual data points. Statistical analysis was conducted using an unpaired two-tailed t-test and the significance level was denoted as (***p < 0.001, ****p < 0.0001, ns > 0.05).

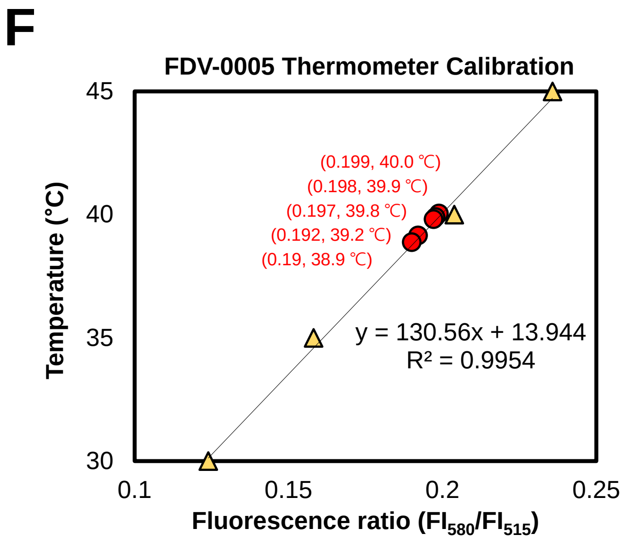

Figure S1 (updated). (**F**) Intracellular temperature calibration was conducted using the FDV-0005 organic thermometer. The FDV-0005 was used at a dilution of 0.05% w/v in 5% glucose solution. Measurements for calibration curve were performed within a temperature range of 30 °C to 45 °C (n = 5). To determine the temperature elevation induced by FCCP, cells were treated with 100 μM FCCP after FDV-0005 incubation for 10 minutes (n = 5). Red dots represent the FCCP-treaded samples.

4 (Reviewer 1-4). The authors tested many conditions to show that FCCP-induced HSF1 foci were due to heat release. However, there is another more direct test that needs to be examined: Maintain the cells at a relatively low temperature, like 32 C, and then add FCCP. In this case, FCCP should still induce most of the other effects in the cell, but now it seems will not be able to increase the cell temperature to >37 C, so this sounds like could be a better control.

Response: Thank you for suggesting another interesting experiment that can further examine whether HSF1 foci formation by FCCP is related to the "temperature". In this experiment, we pre-cooled live cells at 32 °C in a humidified 5% CO2 incubator for 2 hours. Subsequently, we treated the cells with either DMSO or FCCP diluted in DMEM media at 32 °C for 30 minutes to maintain the temperature. Additionally, we treated the cells with FCCP at 37 °C for 30 minutes. As shown in **Figure S4C** and **D** (below), a low population of cells showed HSF1-EGFP foci formation (~3 %) in response to FCCP at 32 °C, whereas much higher proportion of cells exhibited HSF1-EGFP foci in response to FCCP at 37 °C (~22 %). These results indicate that cell incubation conditions at 37 °C are essential for HSF1 foci formation, with additional FCCP-induced mitochondrial thermogenesis. These findings strongly suggest that the formation of HSF1 foci induced by FCCP is dependent on temperature.

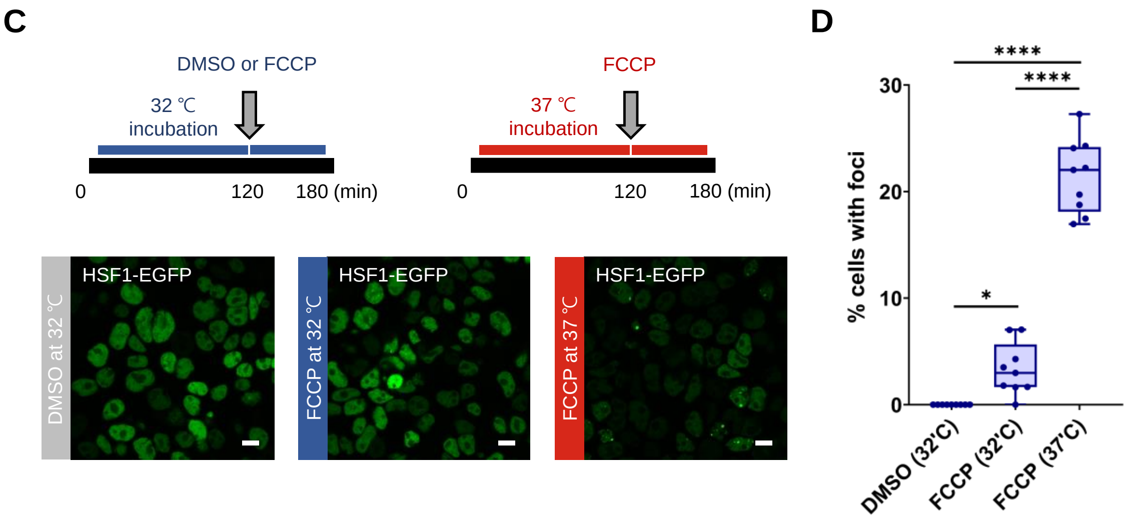

Figure S4 (updated). (**C**) The formation of HSF1-EGFP foci induced by FCCP at 32 °C was visualized by confocal imaging. Cells were pre-cooled at 32 °C in a humidified 5% CO2 incubator for 2 h. FCCP-treated cells at 37 °C were used as positive control. (**D**) Statistics on HSF1-EGFP foci, 450-750 cells were evaluated to obtain the percentage of the cells with foci. Boxes indicate the quartiles, whiskers range from minimal to maximal values, and dots represent individual data points plotted on the box. Statistical analysis was conducted using a one-way analysis of variance (ANOVA) with Tukey's test, and the significance level was denoted as (*$p < 0.05$, ****$p < 0.0001$).

5 (Reviewer 1-5). The authors discuss that in FCCP-treated cells, HSF1 western blot showed slower migrations, and they attributed it to phosphorylation. This discussion is not convincing, e.g., in Fig S3B, no significant size differences are found vs the control. Also, phosphorylation tends to generate smeared bands or multiple bands, unless it is well-defined for a particular state?

Response: Thank you for raising this concern. We agree that slower migration of HSF1 in a western blot result is indirect evidence of phosphorylation but may show relevance, as described in the previous studies[1]. To examine whether the slower migration of HSF1 is due to phosphorylation, we incubated lambda protein phosphatase (λPPase) in the cell lysate sample. λPPase can actively hydrolyze phosphorylated moieties on the HSF1, thus allowing HSF1 to migrate normally. This method has been utilized in the previous report[1] and successfully demonstrated that HSF1 can be hyper-phosphorylated under the heat shock condition. Consequently, we observed that the migration of the HSF1 band induced by FCCP treatment was effectively abolished by the treatment of λPPase (Figure S6A and B, below).
Moreover, we used an antibody specific to phosphorylated HSF1 at serine-326 for direct detection, as this residue is one of the phosphorylation sites triggered by heat shock[1]. Subsequently, we observed a phosphorylated HSF1 band in both FCCP and heat shock conditions. In contrast, there was no signal in the control, menadione and λPPase treated samples (Figure S6C, below). For reviewer's information, we obtained the same lambda protein phosphatase (New England Biolabs, cat. no. P0753S) and phosphorylated HSF1 (phospho-S326) antibody (abcam, cat. no. ab115702) used in the previous study and followed the same incubation protocol (see Methods in SI). These results clearly confirm that FCCP induced phosphorylation of HSF1. We thank the reviewer again for giving us this great opportunity to improve our result in a more convincing way.

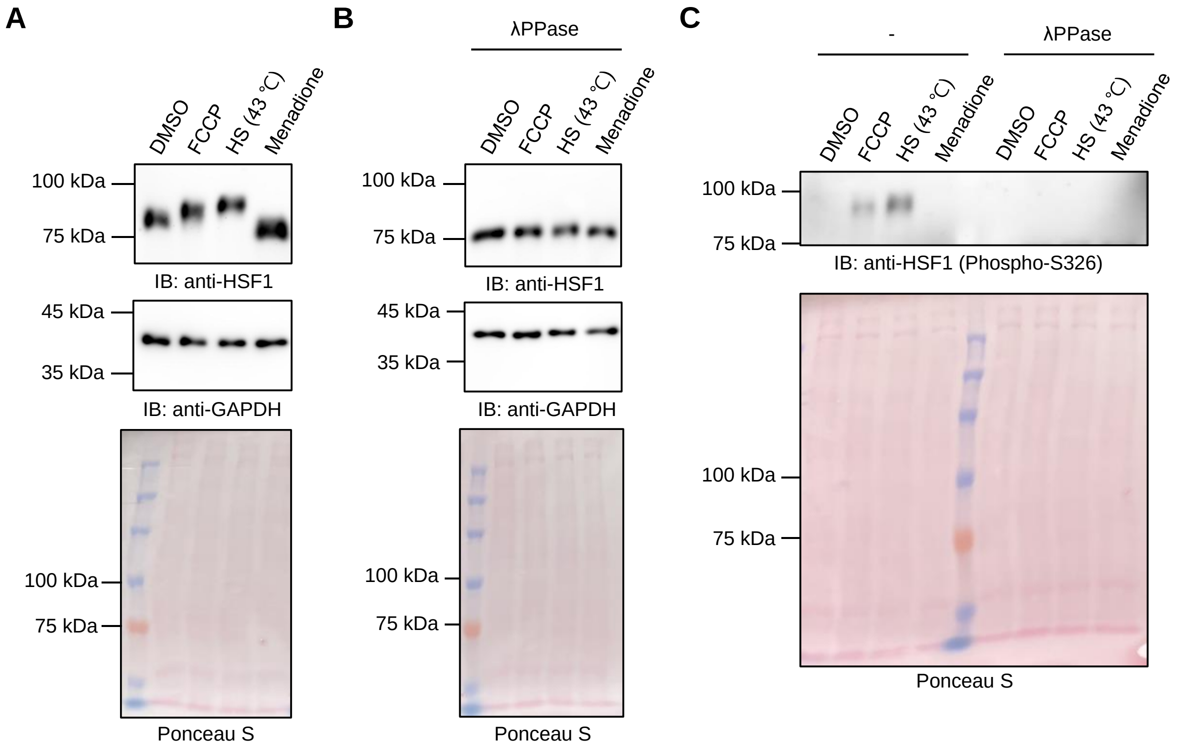

Figure S6 (updated). (**A-B**) Western blot analysis of endogenous HSF1 following treatment with FCCP (100 μM, 1 h) or menadione (30 μM, 30 min) or heat shock (43 °C, 1 h) in MCF10A cells. DMSO-treated cells were used as the control. Lysates were incubated with only buffer condition (**A**) or with lambda protein phosphatase (λPPase) solution (**B**) for 30 minutes before gel running. GAPDH and Ponceau S staining were used as references. (**C**) Detection of phosphorylated HSF1 (phospho-S326) in the same stress conditions used in **A**. Ponceau S staining was used as a reference.

6 (Reviewer 1-6). The discussion of "which leads to which" may be confusing in their discussion of the RNA-seq data. HSF1 is a transcription factor, so its activation could have led to the activation of other genes?

Response: We apologize for the redundancy in our use of the term "activation" in different mechanisms. Regarding the activation of HSF1, the "activation" refers to the structural changes in HSF1 by elevated temperature caused by FCCP-induced thermogenesis or heat shock. These structural changes in HSF1 may lead to its oligomerization (foci formation) or phosphorylation modification by kinase, which are beneficial for its subsequent transcriptional activity in the nucleus. We refer to this state as an activation mode of HSF1. In the context of gene activation, "activation" means the enhanced transcription of the gene regulated by transcription factors such as HSF1. However, we agree that using the same term for different biological events can lead to confusion. Therefore, we have updated this expression with more detailed mechanistic descriptions. We have also updated the discussion section of the RNA-seq results to state: "Our findings suggest that FCCP-mediated heat generation activates HSF1 to bind to heat shock response genes, leading to an increase in the transcriptional level of HSPs, similar to external heat shock conditions." We believe that these revisions address the issue effectively and hope that they are acceptable to the reviewer.

7 (Reviewer 1-7). For the gene expression fold-change data, it may be useful to plot the fold-change in FCCP-treated cells versus heat-shock cells for each gene, then a positive correlation may be identified.

Response: As suggested by the reviewer, we attempted to generate a scatter plot of fold changes in mRNA expression levels for each of the common-gain gene (61 genes) under the FCCP- or heat-shock conditions. As a result (**Figure S8C**, below), $\log_2$ fold change values of FCCP/control and those of Heat shock/control showed positive correlation, with a Pearson coefficient value of 0.57. This relatively positive correlation is expected, as the FCCP treatment (~40 °C) represents a slightly milder heat shock condition compared to the incubation at 43 °C, resulting in different levels of the cellular responses, including transcription levels. We have included this graph and its corresponding data in the Supporting information (**Figure S8C**) and in **Supporting Dataset S1**.

C

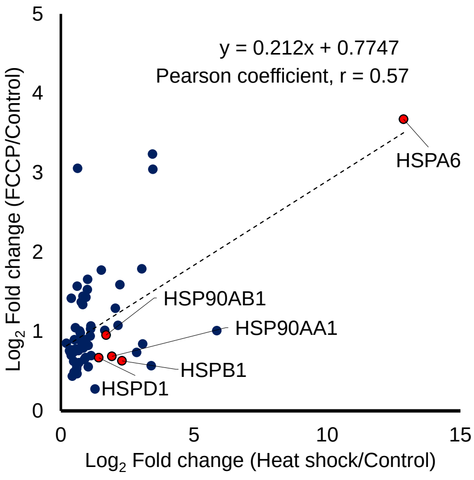

Figure S8C. Scatter plot of mRNA expression level of Common-GAIN genes (61 gene) under the heat shock (x-axis) and FCCP treatment condition (y-axis). In each condition, Log2 fold change values of mRNA expression over the control sample were presented in the graph. Original data for this graph is shown in **Supporting Dataset S1**.

Reviewer #2 (Remarks to Author):

In human cells, mitochondrial dysfunction induces a stress-induced change in gene expression. The heat stress response is part of this reaction. Previous studies have elucidated mechanisms by which mitochondrial proteins (precursor proteins) can trigger reactions in the nucleus. The best-characterized response program is that of C elegans where the transcription factor ATFS-1 is equipped with a mitochondrial signal sequence. Mitochondrial dysfunction prevents the mitochondrial translocation of this factor which therefore ends up in the nucleus to trigger a mitochondrial stress response (Nargund et al. 2012. Science 337, 587-590). Similar response pathways were described in human cells.

In this study, the authors use the well-characterized ionophore FCCP. By uncoupling the proton gradient of the mitochondrial inner membrane, FCCP leads to mitochondrial dysfunction and the increased production of reactive oxygen species (ROS). Uncoupling leads to heat production in cells (shown also in Figure S1) and triggers the heat shock response (Figure 1+3). The authors present some evidence that ROS production is not essential for induction of the heat shock response, which is in line with previous studies. Based on their results, they propose that the production of heat in mitochondria triggers the heat shock response. This is an interesting hypothesis. However, no direct evidence is provided here. Previous studied delineated the stress response pathways in detail. For example, a recent publication by **Munch (Sutandy et al. 2023 Nature 618, 849-854)** showed that the binding of non-imported mitochondrial proteins to the HSP40 protein DNAJA1 is crucial for the response to mitochondrial dysfunction (including the HSR). The relevance of mitochondrial precursor proteins was also reported before in many other studies. FCCP induces the accumulation of mitochondrial precursor proteins in the cytosol. Rhee and coworkers now suggest that thermogenesis, but not accumulating precursor proteins, trigger the heat shock response. However, as it stands, evidence for this claim is missing.

We thank the reviewer for insightful comments which are very helpful for improvement of our works. We conducted several additional experiments according to the reviewer's suggestion and we hope that our improved manuscript with additional results can be acceptable to the reviewer.

Specific problems:

1 (Reviewer 2-1). The authors need to study conditions at which the effect on heat production can be “uncoupled” from that on protein import.

Response: Thank you for addressing this concern. The comment is crucial as FCCP is reported to induce the loss of membrane potential, leading to disturbances in the mitochondrial protein import by stalling proteins at both the TOM and TIM complexes, as shown in the previous studies. As the reviewer suggested, we utilized gamitrinib-triphenylphosphonium (GTPP) to determine whether GTPP induces HSF1 foci formation under proteotoxic stress because Dr. Munch’s lab showed GTPP induced HSF1 translocation to the nucleus via biochemical assay (i.e. fractionation) in their recent study[2] as noted by the reviewer. However, this study did not

characterize the imaging experimental results of HSF1 foci formation. Prior to the experiment, we obtained the GTPP molecule from Prof. Byoung Heon Kang, who first developed it[3]. We also checked the purity of GTPP using high resolution mass spectrometry (HRMS, **Figure R1, below**). After confirmation, we treated our cells with GTPP at the same concentration and incubation time as reported. Consequently, we observed fewer numbers of HSF1 foci formed after GTPP treatment compared to the FCCP-treated cells (**Figure S3G-H, below**). This result indicates that the formation of HSF1 foci is less sensitive to the proteotoxicity than heat.

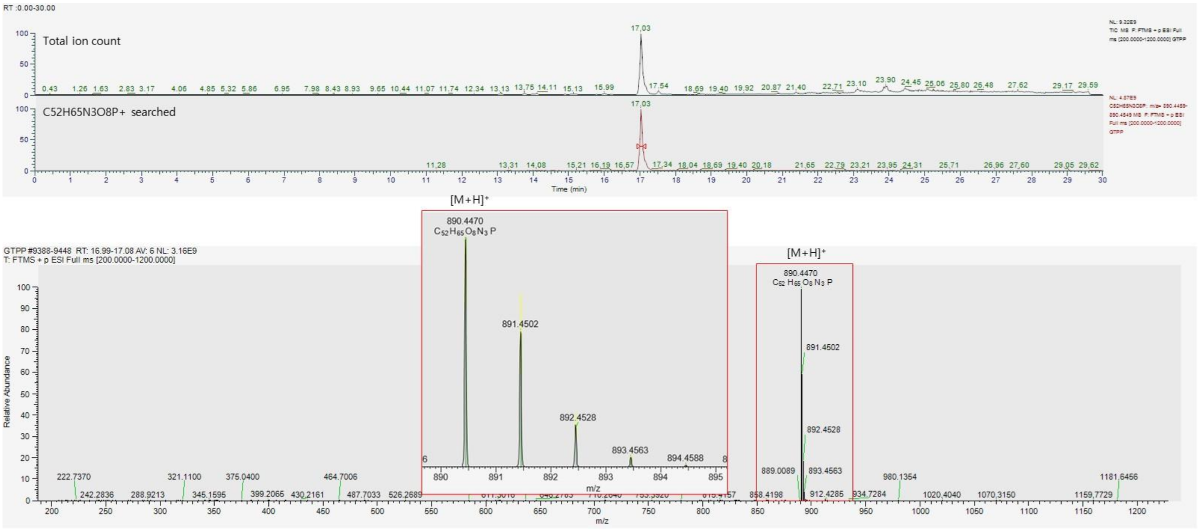

Figure R1. High resolution mass spectrometry result of the GTPP molecule (MW 890.4504., detected ([M+H]+, m/z): 890.4470). LC-HRMS (Orbitrap Exploris 120 coupled with Vanquish VC-P10-A (Thermofisher Scientific, USA) equipped with Hypersil Gold C18 Selectivity 4.6 x 150 mm, 5 um (Thermofisher, USA) was utilized for the mass characterization.

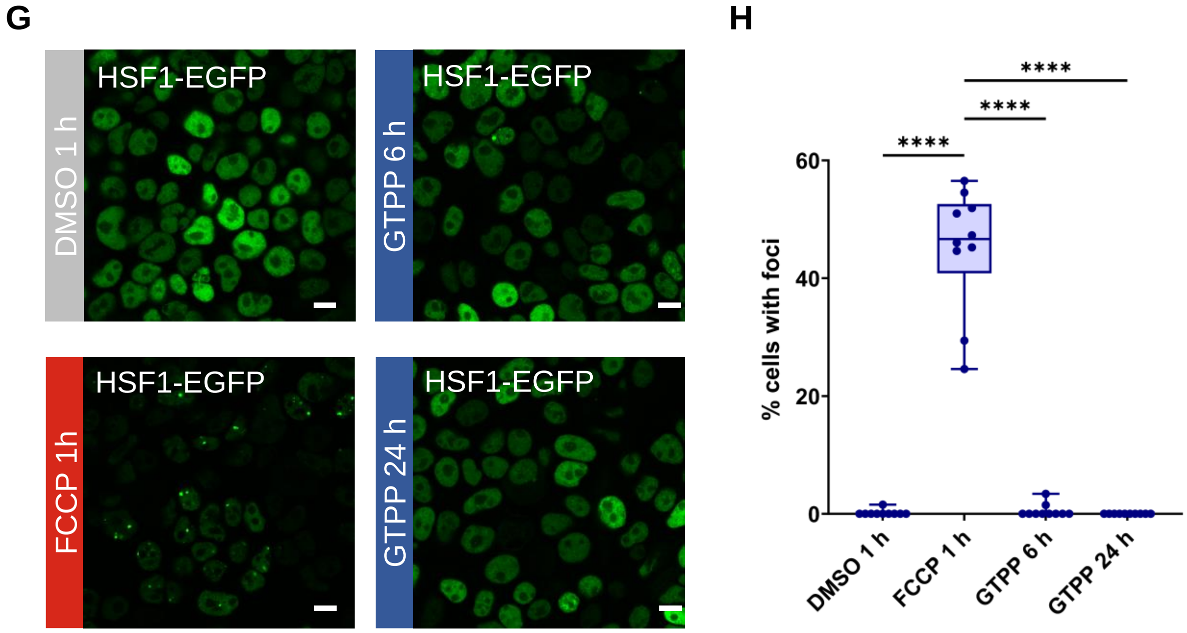

Figure S3 (updated). (**G**) Confocal images of HSF1-EGFP foci formation were obtained after treatment with either DMSO or FCCP (100 μM) for 1 h or GTPP (10 μM) for 6 or 24 h. (**H**) Quantification of percentage of the cells with foci was performed by evaluating 500-640 cells. Boxes indicate the quartiles, whiskers range from minimal to maximal values, and dots represent individual data points plotted on the box. Statistical analysis was conducted using a one-way analysis of variance (ANOVA) with Tukey's test, and the significance level was denoted as (****$p < 0.0001$).

Furthermore, we monitored whether the mitochondrial import process of endogenous HSPD1 or the mitochondrial targeting sequence (MTS, from COX4)-myc-dsRed were perturbed by our FCCP treatment condition (100 μM FCCP for 1 hr). As shown in **Figure S3A** and **B** below, fluorescence imaging results indicate that our FCCP treatment condition did not induce cytosolic accumulation of either MTS-myc-dsRed or HSPD1 in the cells. Additionally, the uncleaved precursor form of endogenous HSPD1 was not detected in Western blotting result (**Figure S3C** and **D**). These results indicate that the level of accumulated mitochondrial targeted proteins in cytosol might be negligible in our conditions, which might be too short to induce the proteotoxic effect in the cells. Based on these results and those results presented in the subsequent response (cycloheximide treatment), we could suggest that our observed HSF1 foci formation by FCCP could not be induced by the proteotoxic effect of this chemical. Our other experimental results related to temperature (e.g. intracellular temperature measurement by FDV and ERthermoAC, external temperature changes) support this conclusion, indicating that HSF1's foci formation by FCCP in our conditions has a strong correlation with intracellular temperature changes primarily induced by mitochondrial thermogenesis.

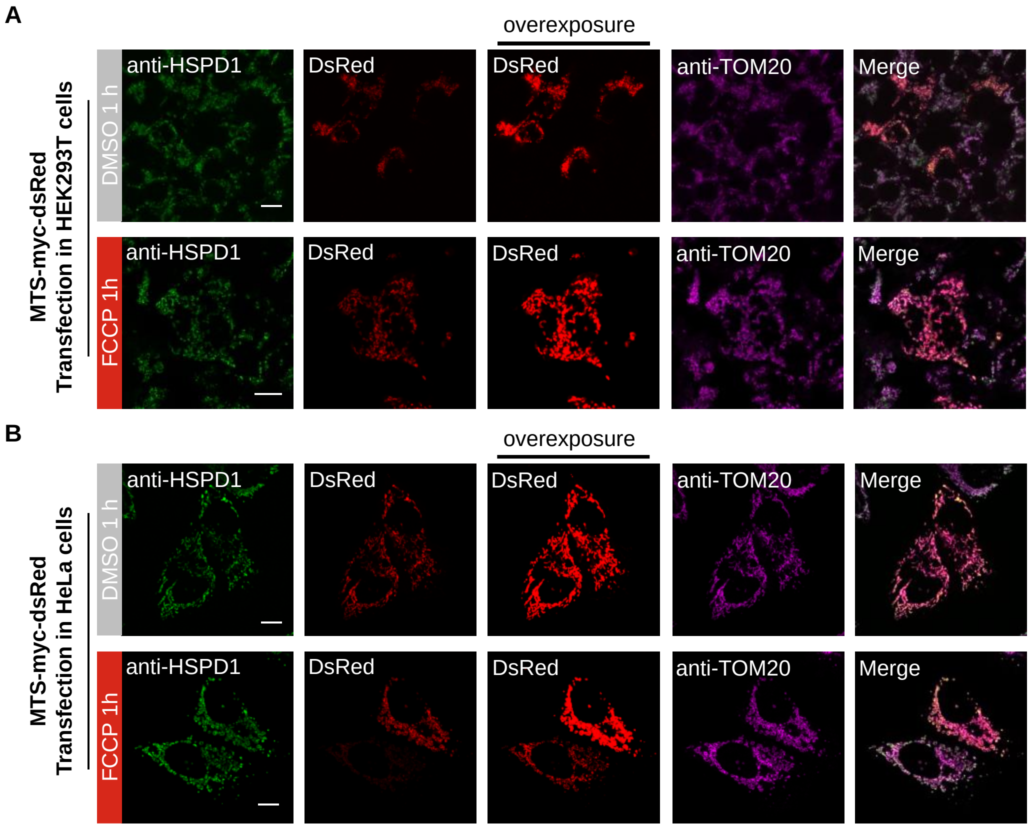

Figure S3 (updated). Subcellular localization of transiently expressed MTS-myc-dsRed and endogenous HSPD1 was visualized in HEK293T (**A**) and HeLa cells (**B**) by immunofluorescence with anti-Myc and anti-HSPD1 antibodies. Scale bar = 10 µm.

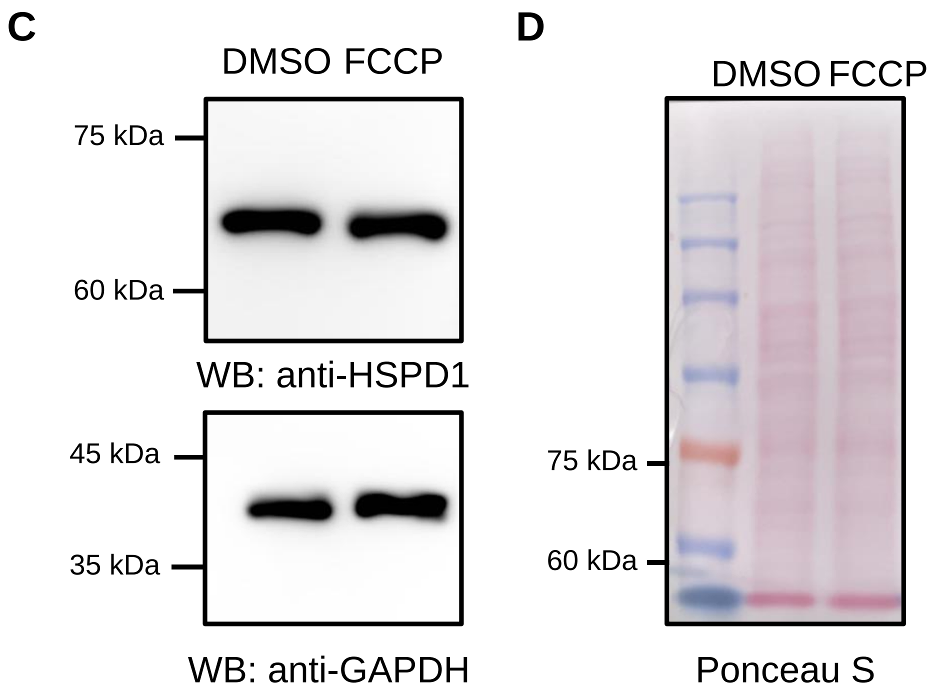

Figure S3 (updated). (**C**) Western blot analysis of endogenous HSPD1 under the DMSO (control) or FCCP (100uM, 1hr)-treated sample using an anti-HSPD1 antibody. (**D**) Ponceau staining of the same membrane in (**C**) was used as reference.

2 (Reviewer 2-2). Along the same lines: can the authors repress the synthesis of mitochondrial precursor proteins so that their accumulation does not trigger the HSR upon FCCP treatment?

Response: Thank you for suggesting this experiment. To repress the newly synthesized protein, including mitochondrial precursor proteins, we utilized cycloheximide (CHX) and puromycin. For this experiment, we pre-treated the cells with 35 µM CHX or 35 µM puromycin before FCCP treatment. Then, we added DMSO or FCCP to the media containing CHX and puromycin. As a result, we found that HSF1 foci were effectively formed by FCCP treatment even though the process of protein synthesis is blocked by CHX or puromycin (**Figure S3E** and **F** below). This experimental result may support the notion that HSF1 foci formation induced by FCCP is an independent process from protein accumulation.

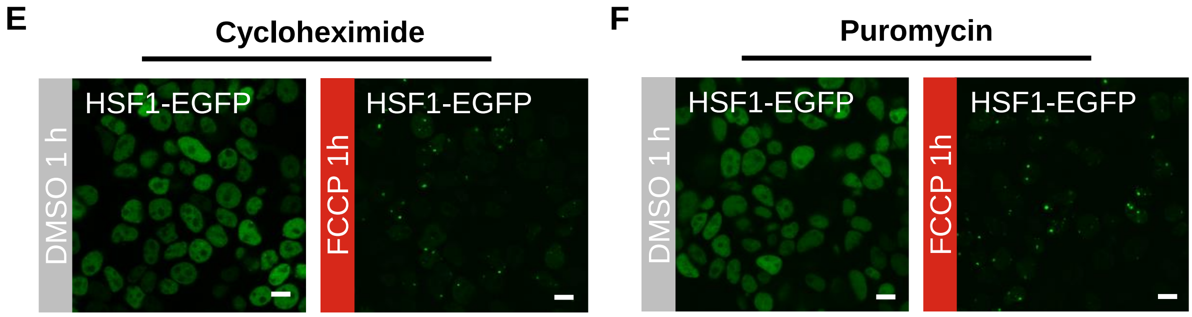

Figure S3 (updated). Foci formation of HSF1-EGFP in FCCP-treated cells under protein synthesis blockade by cycloheximide (**E**) and puromycin (**F**) was observed. Cycloheximide (35 μM) and puromycin (35 μM) were pre-treated for 2 h followed by addition of DMSO or FCCP (100 μM) for 1 h. Scale bar = 10 μm.

3 (Reviewer 2-3). In general, FCCP is a very harsh treatment and many studies in the context of PINK-Parkin showed that the FCCP-induced effects are more extreme than those detected under physiological conditions. Thus, under the normal conditions of mitochondrial function or dysfunction, does mitochondrial thermogenesis for the HSR really matter? It seems unlikely to this referee that outside of brown adipose tissue cells, heat production from mitochondria will be of any physiological relevance.

Response: We appreciate engaging in the fundamental question of our study. We agree that FCCP treatment is a harsh condition; however, it is widely used to measure the maximal mitochondrial oxygen consumption rate of various mammalian cells in Seahorse and previous studies have utilized FCCP for the measurement of increased intracellular temperature of live cells[4,5,6]. For these reasons, we primarily utilized FCCP treatment as an experimental setting in our study. Importantly, we also confirmed that BAM15, a mild type of protonophores commonly used in *in vivo* study[7], was sufficient to from HSF1 foci (**Figure S5**, below). Unfortunately, as Parkin-PINK pathways seemed not conserved in all mammalian cellular systems[8], we were not able to determine whether Parkin-PINK is activated upon FCCP/BAM15 treatments. In support of this expectation, our mitochondria-focused spatial proteome mapping results using same cellular system (i.e. HEK293T-Rex cell line) revealed that BAM15 treatment failed to uncover PINK1-Parkin related proteins (unpublished results).

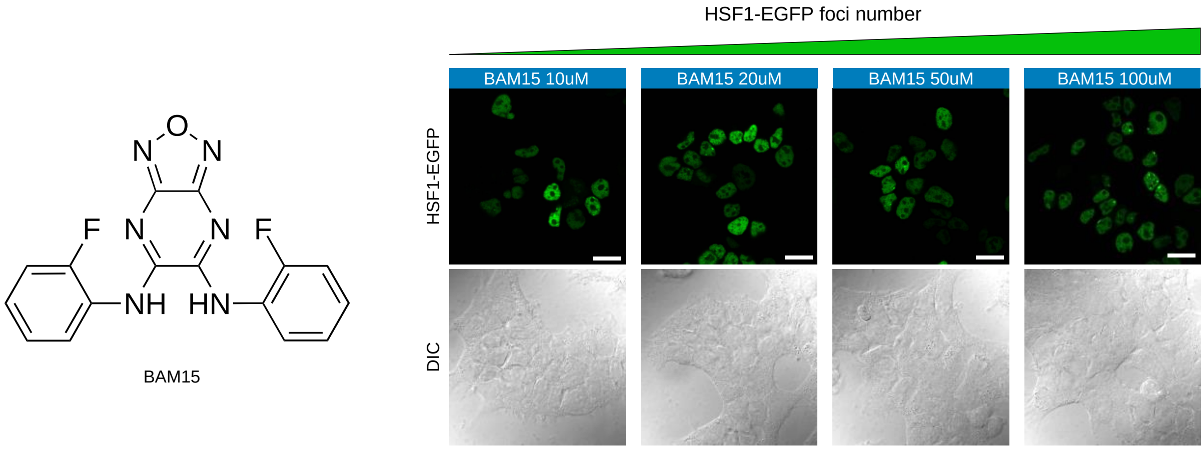

Figure S5. (**D**) Confocal images of HSF1-EGFP foci formation in HEK293T expressing HSF1-

EGFP under treatment with various BAM15 concentrations (10, 20, 50, and 100 µM for 1 h incubation). Scale bar 20 µm.

As the reviewer noted, it would be very important to verify the physiological meaning of our findings. We believe that our findings can make two important messages for current understanding: mitochondrial thermogenesis and HSF1 activation. To the best of our knowledge, our study is the first to connect the exothermic enthalpy change of this oxygen reduction consumption reaction ($O_2 + 4H^+ + 4e^- \rightarrow 2H_2O$, $\Delta H_f° = -285$ kJ/mol) to the mitochondrial thermogenesis. We suggest that this reaction is likely the primary thermogenic process in mitochondria. We validated that every component (i.e., protons, oxygen, and electrons) in this reaction is crucial for sufficient mitochondrial thermogenesis and subsequent upregulation of HSF1. Since the oxygen consumption rate is considered a reliable indirect measurement standard for thermogenic events in the metabolism research field[9], we believe that our theorem may be accepted in the field.

Moreover, we anticipate that our ORR-driven thermogenesis model can provide a new connection between mitochondrial respiration and mitochondrial thermogenesis, potentially applicable to all actively respiring cells. Accumulating evidence suggests that not only brown adipose tissue but also various tissue-originated mammalian cells, including cancer cells, exhibit active mitochondrial respiration activities[10-13]. Thus, we expect that these actively respiring cells may experience elevated intracellular temperature and activate the HSF1 signaling pathway, possibly due to active ORR-driven mitochondrial thermogenesis.

In support of this notion, we conducted preliminary experiments to investigate whether FCCP induces HSF1 foci formation in various immortalized cell lines. As shown in **Figure R2** (below), HeLa (cervical carcinoma), HepG2 (hepatocellular carcinoma), U87MG (glioblastoma), HMC3 (immortalized microglia), MGCL (immortalized microglia), U2OS (osteosarcoma), NCI-H 1437 (lung adenocarcinoma) exhibited HSF1 foci under FCCP treatment. Conversly, N2A (neuroblastoma cells), MEF (mouse embryonic fibroblast), TM3 (Leydig cell), WI-38 (fibroblast-like fetal lung cell), H295R (adrenal gland) did not display any HSF1 foci (**Figure R3**).

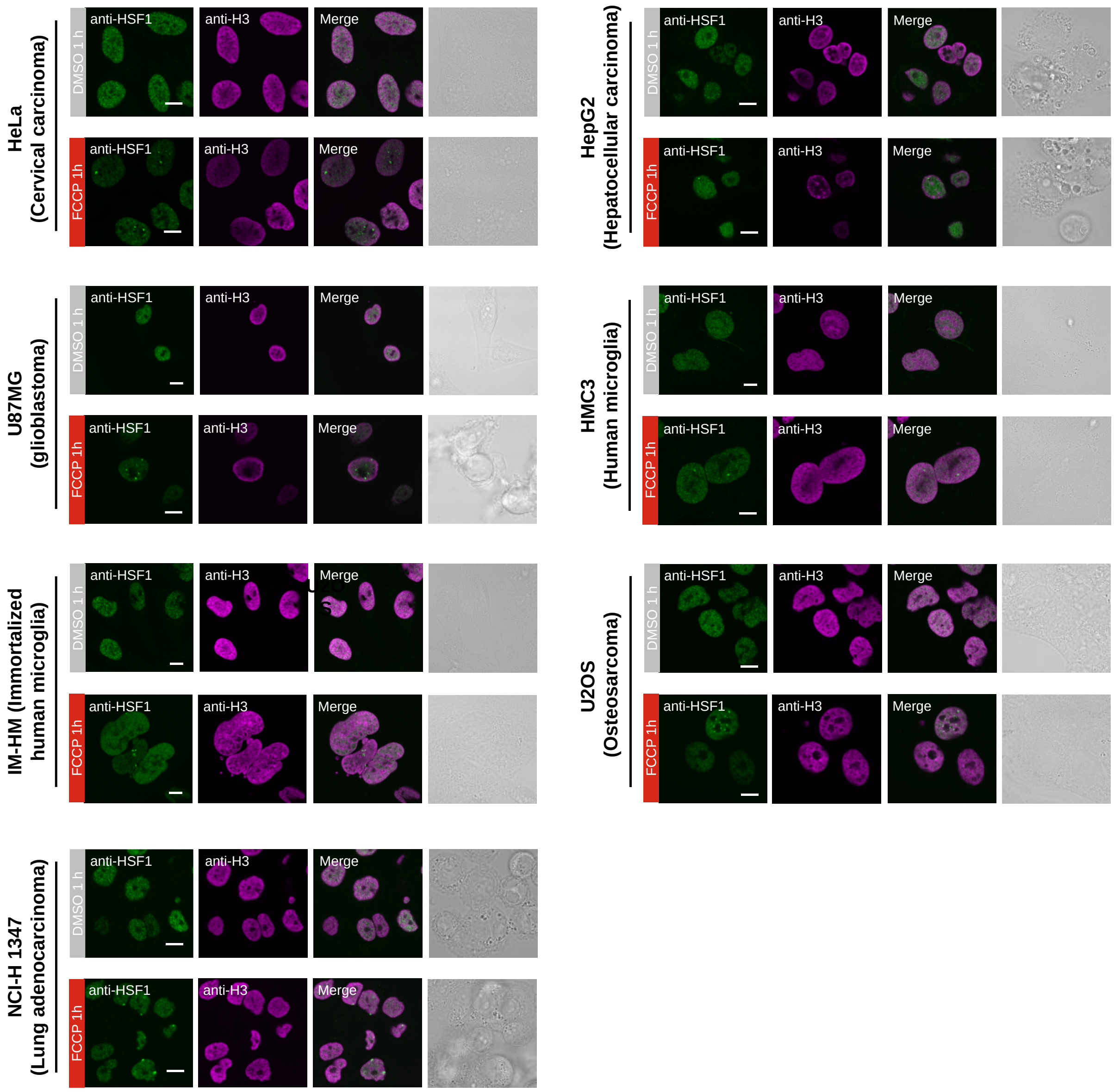

Figure R2. Observation of the formation of endogenous HSF1 foci by immunofluorescence in various tissue-originated cell lines.

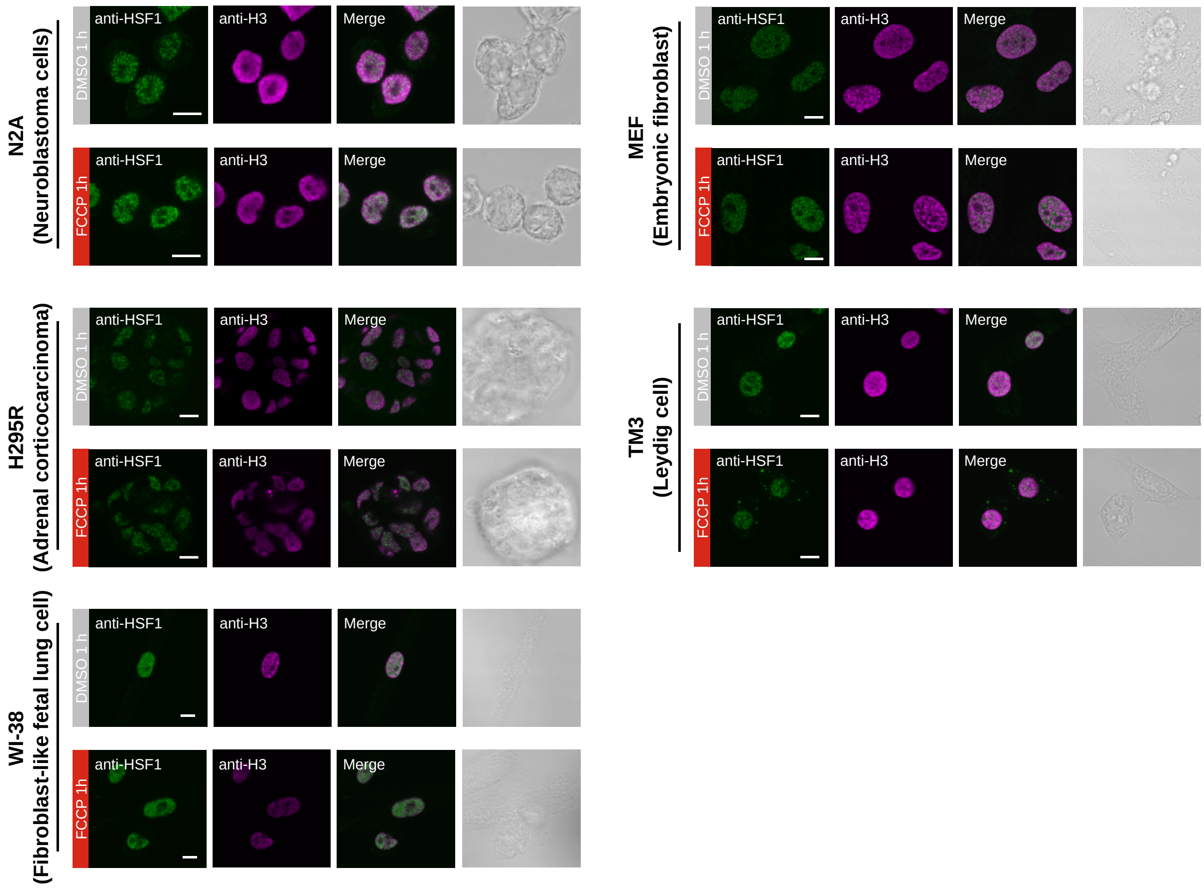

Figure R3. Observation of the formation of endogenous HSF1 foci by immunofluorescence in various cell lines.

Although these results need further characterization, including mitochondrial oxygen consumption rate measurement, our previous intracellular temperature measurements using ERthermAc (ETAC, **Figure S1C, D**, below) showed that FCCP increased the intracellular temperature of Hela and HepG2 cells, which is in good agreement with HSF1 foci formations in those cells (**Figure R2**). We also speculated that certain cells may develop a more "temperature-sensitive" HSF1 foci formation system that can make a prompt response upon mitochondrial thermogenesis. We derived this idea from our preliminary data analysis of the differential HSF1 effect on various tissue-originated cell proliferations (**Figure R4**, below). Since HSF1 foci formations were observed in several aggressive tumors of human patients[14,15], it might be intriguing to further investigate this mito-ORR and HSF1 axis or mitochondrial heat signaling in the cancer research field.

We thank the reviewer again for allowing us room to think about the physiological meaning of our findings. We have added our speculation of how our findings can help improve the current understanding of mitochondrial thermogenesis and HSF1 signaling in the Discussion part of the revised manuscript.

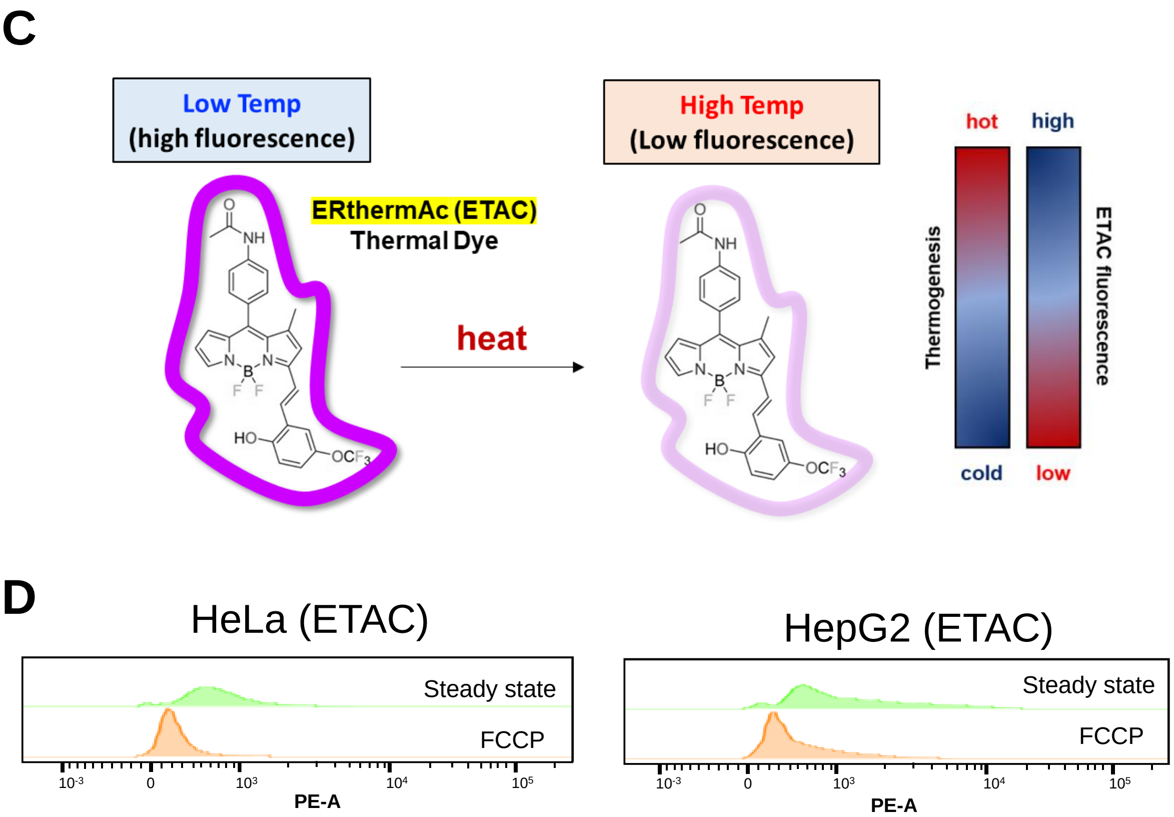

Figure S1. (**C**) Schematic illustration of temperature measurement by ETAC. (**D**) ETAC fluorescence intensity after FCCP (100 µM, 1 h) treatment. Decreased ETAC fluorescence intensity under the indicates increased local temperature at the ER membrane. Flow cytometry analysis results of ETAC fluorescent signal under the two conditions (steady-state or 100 µM FCCP, 1 h), in HeLa and HepG2 cell lines. Cell count is shown on the y-axis, while fluorescent signal intensity (PE-A) is shown on the x-axis.

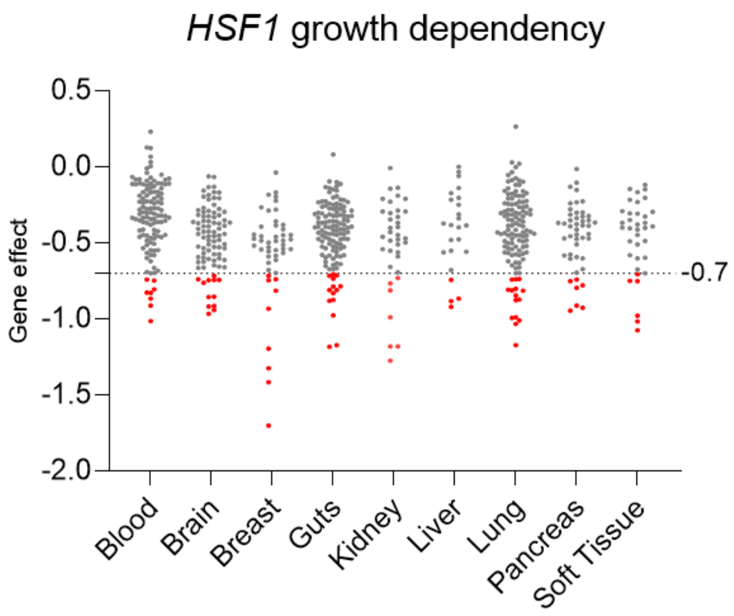

Figure R4. A scatter plot showing the differential HSF1 effect on the cell growth in various tissues. Each dot represents an individual cell line. The gene effect scores were obtained from DepMap Public 23Q4+Score (Chronos method) provided by the DepMap portal[16]. Scores below -0.7, which corresponds to approximately the bottom 15%, were marked in red. The scores for nonessential genes tend towards 0 or above, while the scores for essential genes tend towards negative values.

References

1. Fujimoto, M.; Takii, R.; Matsumoto, M.; Okada, M.; Nakayama, K. I.; Nakato, R.; Fujiki, K.; Shirahige, K.; Nakai, A., HSF1 phosphorylation establishes an active chromatin state via the TRRAP-TIP60 complex and promotes tumorigenesis. *Nat Commun* **2022,** *13* (1), 4355.

2. Sutandy, F. X. R.; Gößner, I.; Tascher, G.; Münch, C., A cytosolic surveillance mechanism activates the mitochondrial UPR. *Nature* **2023,** *618* (7966), 849-854.

3. Kang, B. H.; Plescia, J.; Song, H. Y.; Meli, M.; Colombo, G.; Beebe, K.; Scroggins, B.; Neckers, L.; Altieri, D. C., Combinatorial drug design targeting multiple cancer signaling networks controlled by mitochondrial Hsp90. *J Clin Invest* **2009,** *119* (3), 454-64.

4. Homma, M.; Takei, Y.; Murata, A.; Inoue, T.; Takeoka, S., A ratiometric fluorescent molecular probe for visualization of mitochondrial temperature in living cells. *Chem Commun (Camb)* **2015,** *51* (28), 6194-7.

5. Nakano, M.; Arai, Y.; Kotera, I.; Okabe, K.; Kamei, Y.; Nagai, T., Genetically encoded ratiometric fluorescent thermometer with wide range and rapid response. *PLoS One* **2017,** *12* (2), e0172344.

6. Sugimura, T.; Kajimoto, S.; Nakabayashi, T., Label-Free Imaging of Intracellular Temperature by Using the O-H Stretching Raman Band of Water. *Angew Chem Int Ed Engl* **2020,** *59* (20), 7755-7760.

7. Alexopoulos, S. J.; Chen, S. Y.; Brandon, A. E.; Salamoun, J. M.; Byrne, F. L.; Garcia, C. J.; Beretta, M.; Olzomer, E. M.; Shah, D. P.; Philp, A. M.; Hargett, S. R.; Lawrence, R. T.; Lee, B.; Sligar, J.; Carrive, P.; Tucker, S. P.; Philp, A.; Lackner, C.; Turner, N.; Cooney, G. J.; Santos, W. L.; Hoehn, K. L., Mitochondrial uncoupler BAM15 reverses diet-induced obesity and insulin resistance in mice. *Nat Commun* **2020,** *11* (1), 2397.

8. Villa, E.; Marchetti, S.; Ricci, J. E., No Parkin Zone: Mitophagy without Parkin. *Trends Cell Biol* **2018,** *28* (11), 882-895.

9. Bokhari, M. H.; Halleskog, C.; Åslund, A.; Boulet, N.; Casadesús Rendos, E.; de Jong, J. M. A.; Csikasz, R.; Amri, E.-Z.; Shabalina, I.; Bengtsson, T., Isothermal microcalorimetry measures UCP1-mediated thermogenesis in mature brite adipocytes.

Communications Biology **2021,** *4* (1), 1108.
10. Martin, S. D.; McGee, S. L., A systematic flux analysis approach to identify metabolic vulnerabilities in human breast cancer cell lines. *Cancer & Metabolism* **2019,** *7* (1), 12.
11. Dier, U.; Shin, D.-H.; Hemachandra, L. P. M. P.; Uusitalo, L. M.; Hempel, N., Bioenergetic Analysis of Ovarian Cancer Cell Lines: Profiling of Histological Subtypes and Identification of a Mitochondria-Defective Cell Line. *PLOS ONE* **2014,** *9* (5), e98479.
12. Chrétien, D.; Bénit, P.; Ha, H.-H.; Keipert, S.; El-Khoury, R.; Chang, Y.-T.; Jastroch, M.; Jacobs, H. T.; Rustin, P.; Rak, M., Mitochondria are physiologically maintained at close to 50 °C. *PLOS Biology* **2018,** *16* (1), e2003992.
13. Wagner, B. A.; Venkataraman, S.; Buettner, G. R., The rate of oxygen utilization by cells. *Free Radic Biol Med* **2011,** *51* (3), 700-12.
14. Gaglia, G.; Rashid, R.; Yapp, C.; Joshi, G. N.; Li, C. G.; Lindquist, S. L.; Sarosiek, K. A.; Whitesell, L.; Sorger, P. K.; Santagata, S., HSF1 phase transition mediates stress adaptation and cell fate decisions. *Nature Cell Biology* **2020,** *22* (2), 151-158.
15. Mendillo, M. L.; Santagata, S.; Koeva, M.; Bell, G. W.; Hu, R.; Tamimi, R. M.; Fraenkel, E.; Ince, T. A.; Whitesell, L.; Lindquist, S., HSF1 drives a transcriptional program distinct from heat shock to support highly malignant human cancers. *Cell* **2012,** *150* (3), 549-562.
16. Dempster, J. M.; Boyle, I.; Vazquez, F.; Root, D. E.; Boehm, J. S.; Hahn, W. C.; Tsherniak, A.; McFarland, J. M., Chronos: a cell population dynamics model of CRISPR experiments that improves inference of gene fitness effects. *Genome Biol* **2021,** *22* (1), 343.

oc-2023-015892.R2

Name: Peer Review Information for "Mitochondrial thermogenesis can trigger heat-shock response in the nucleus"

Second Round of Reviewer Comments

Reviewer: 2

Comments to the Author

The referees addressed most of my critical points. I therefore now support publication of this study. However, I think the title should be changed. This study is based on a very artificial situation (FCCP treatment in brown adipose tissue). It remains unclear (and actually seems unlikely) that mitochondrial thermogenesis is of relevance under physiological conditions, particularly in other tissues. Therefore, the authors should change the title, e.g. to 'Mitochondrial thermogenesis can trigger heat-shock response in the nucleus'

Reviewer: 1

Comments to the Author

The authors have done a good job with the revision, and the quality of the manuscript is further improved. I recommend publication.

Author's Response to Peer Review Comments:

Reviewers' Comments

Reviewer #2 (Remarks to Author)

Comments:

The referees addressed most of my critical points. I therefore now support publication of this study. However, I think the title should be changed. This study is based on a very artificial situation (FCCP treatment in brown adipose tissue). It remains unclear (and actually seems unlikely) that mitochondrial thermogenesis is of relevance under physiological conditions, particularly in other tissues. Therefore, the authors should change the title, e.g. to 'Mitochondrial thermogenesis can trigger heat-shock response in the nucleus'

Response: We have changed the title of the manuscript to 'Mitochondrial thermogenesis can trigger heat-shock response in the nucleus', as suggested by the reviewer. We appreciate the reviewer for their extremely helpful comments and constructive suggestions for improving our manuscript throughout the entire revision process.

Reviewer #1 (Remarks to Author)

Comments:

The authors have done a good job with the revision, and the quality of the manuscript is further improved. I recommend publication.

Thank you for your kind comment. We appreciate that our manuscript has been logically and statistically improved based on the reviewer's comments throughout the entire revision process.
